# MyD88 signaling in dendritic cells and the intestinal epithelium controls immunity against intestinal infection with *C*. *rodentium*

**DOI:** 10.1371/journal.ppat.1006357

**Published:** 2017-05-16

**Authors:** Christin Friedrich, Panagiota Mamareli, Sophie Thiemann, Friederike Kruse, Zuobai Wang, Bernhard Holzmann, Till Strowig, Tim Sparwasser, Matthias Lochner

**Affiliations:** 1 Institute of Infection Immunology, TWINCORE, Centre for Experimental and Clinical Infection Research; a joint venture between the Medical School Hannover (MHH) and the Helmholtz Centre for Infection Research (HZI), Hannover, Germany; 2 Institute of Medical Microbiology and Hygiene, University of Freiburg, Freiburg Medical Centre, Freiburg, Germany; 3 Microbial Immune Regulation, Helmholtz Centre for Infection Research, Braunschweig, Germany; 4 Department of Surgery, Technische Universität München, Munich, Germany; University of São Paulo FMRP/USP, BRAZIL

## Abstract

MyD88-mediated signaling downstream of *Toll*-like receptors and the IL-1 receptor family is critically involved in the induction of protective host responses upon infections. Although it is known that MyD88-deficient mice are highly susceptible to a wide range of bacterial infections, the cell type-specific contribution of MyD88 in protecting the host against intestinal bacterial infection is only poorly understood. In order to investigate the importance of MyD88 in specific immune and nonimmune cell types during intestinal infection, we employed a novel murine knock-in model for MyD88 that enables the cell type-specific reactivation of functional MyD88 expression in otherwise MyD88-deficient mice. We report here that functional MyD88 signaling in CD11c^+^ cells was sufficient to activate intestinal dendritic cells (DC) and to induce the early group 3 innate lymphoid cell (ILC3) response as well as the development of colonic Th17/Th1 cells in response to infection with the intestinal pathogen *C*. *rodentium*. In contrast, restricting MyD88 signaling to several other cell types, including macrophages (MO), T cells or ILC3 did not induce efficient intestinal immune responses upon infection. However, we observed that the functional expression of MyD88 in intestinal epithelial cells (IEC) also partially protected the mice during intestinal infection, which was associated with enhanced epithelial barrier integrity and increased expression of the antimicrobial peptide RegIIIγ and the acute phase protein SAA1 by epithelial cells. Together, our data suggest that MyD88 signaling in DC and IEC is both essential and sufficient to induce a full spectrum of host responses upon intestinal infection with *C*. *rodentium*.

## Author summary

MyD88-dependent signaling pathways play a critical role in the protective immune response during intestinal infections. However, the significance of MyD88-mediated signaling in specific intestinal immune and nonimmune cell types for the activation of the early innate, adaptive and epithelial host responses upon infection remains poorly understood. Using a novel knock-in mouse model for MyD88, we report here that MyD88 signaling in CD11c^+^ dendritic cells (DC) is sufficient to activate RORγt^+^ group 3 innate lymphoid cells (ILC3) as well as Th17/Th1 cells in response to infection with *C*. *rodentium*. In contrast, restricting functional MyD88 signaling to several other immune cell types, including macrophages (MO), T cells and ILC3 did not result in intestinal immunity, while expression of MyD88 in intestinal epithelial cells (IEC) mainly enhanced epithelial barrier integrity. Together, our data suggest that MyD88 signaling in DC and IEC is both essential and sufficient to induce a full spectrum of host responses upon intestinal infection with *C*. *rodentium*.

## Introduction

The ability to mount specific inflammatory responses is pivotal to the fight against pathogens. The non-invasive attaching-effacing bacterial pathogen *Citrobacter rodentium* has been well appreciated as a model to study the processes that lead to the activation of innate and adaptive components of the intestinal immune system. During the early phase of infection, the cytokine IL-22 is essential to confer host protection [1] and RORγt-expressing group 3 innate lymphoid cells (ILC3) have been identified as a critical cellular source of this cytokine [2, 3]. Binding of IL-22 to the IL-22 receptor expressed on the intestinal epithelium can have multiple effects, including the enhanced secretion of antimicrobial peptides such as RegIIIγ [1], increased production of mucus [4] as well as the induction of processes that promote survival and enhanced proliferation of intestinal epithelial cells (IEC) [5–7]. Thus, the activity of IL-22 on the epithelium is crucial for protecting the intestinal barrier integrity during infection and supporting the induction of tissue repair and regeneration.

In addition, infection with *C*. *rodentium* induces a massive T cell-mediated adaptive response that is necessary to clear the pathogen at the later stages of infection, but also causes much of the colonic immunopathology and colitis-like disease symptoms that occur during the infection [8]. Both IFN-γ-producing Th1 cells and IL-22-secreting Th22 cells have been reported to be critical effectors of the host response [9–11]. Additionally, a strong Th17 cell response is induced upon infection [12] and mice that lack the Th17 cytokines IL17A/F showed an enhanced susceptibility towards infection with *C*. *rodentium* [13]. This phenotype was associated with a reduced induction of antimicrobial β-defensins in the colon, suggesting that IL-17 may act mainly by enhancing the intestinal barrier function. This is in agreement with data suggesting that IL-17 can directly affect gut permeability by regulating the organization of tight junctions in intestinal epithelial cells [14, 15]. Importantly, interfering with the proper induction of IL-17/IFN-γ-producing T cells following *C*. *rodentium* infection leads to reduced inflammatory pathology in the colon, but at the same time enhances systemic pathogen dissemination and increases mortality, together highlighting the importance of Th17/Th1 cells for both pathogen clearance and the inflammation-associated colitis phenotype [16].

Intestinal CD11c^+^ mononuclear phagocytes (MNP) that comprise bona-fide dendritic cells (DC) as well as macrophages (MO) play an important role in the induction of innate and adaptive immune responses upon infection with *C*. *rodentium*. Production of IL-23 by MNP is crucial for both the early innate as well as the T cell-mediated host response, since it directly stimulates IL-22-production by ILC3 and also contributes to the development of Th17 cells in the gut [9, 12, 17–19]. Studies that have addressed the relative contribution of DC and MO to the immune response, however, came to differing conclusions. CD11b^+^CD103^+/-^ DC, representing the conventional DC2 subset, have been implicated in the induction of Th17 cell responses under steady state conditions [20, 21] and in the IL-23-dependent activation of innate IL-22 production [19, 22], although the role of CD11b^+^CD103^+^ DC at least in the latter process has recently been questioned [23]. Other publications have suggested that CX_3_CR1^+^ MNP, which predominantly differentiate from monocyte precursors [24], provide essential support in the induction of the IL-22-mediated innate response upon infection with *C*. *rodentium* [17, 18].

It is well established that the activation of myeloid differentiation primary response gene 88 (MyD88)-mediated signaling downstream of *Toll-like* receptors (TLR) and the IL-1 receptor family is indispensable for inducing both protective host responses and immunopathology upon intestinal infection with *C*. *rodentium* [25–29]. MyD88 signaling, however, can be induced in several intestinal cell types, including IEC, MNP as well as ILC and T cells, through either TLR or IL-1 receptor family activation. Bone marrow transfer approaches indicated that MyD88 signaling in both the hematopoietic and non-hematopoietic compartments contributes to the infection-associated response [29]. However, immune cell types such as ILC have proven to be radio-resistant [30], thus making the interpretation of such data difficult. The consequences of MyD88 signaling in different intestinal cell types upon initiation of the innate and adaptive responses therefore still remain largely undefined.

In the present study we make use of a recently generated knock-in mouse model for cell type-specific expression of MyD88 [31]. In this model, only specifically targeted cell types express functional MyD88, while all other cells remain MyD88-deficient. It is thus possible to unambiguously dissect the direct impact of functional MyD88-mediated signaling in specific cell types, and to determine the consequences for the activation of the different processes of the host response upon infection. Using this novel approach, we demonstrate that functional MyD88 in CD11c^+^ MNP is sufficient to activate ILC3 and to protect mice during the early phase of infection with *C*. *rodentium*. MyD88 signaling in CD11c^+^ MNP activated the expression of proinflammatory cytokines in colonic DC, including IL-6, IL-1β and IL-23, which resulted in a restoration of the Th17/Th1 cell response. In contrast, both MO- as well as ILC3- and T cell-intrinsic MyD88 signaling alone was not sufficient to induce a significant innate or adaptive immune response. Importantly, our experiments also demonstrate a critical contribution of IEC-intrinsic MyD88 signaling for maintaining the epithelial barrier during infection, together highlighting the importance of MyD88 in both DC and IEC for the induction of the host defense in the intestine.

## Results

### Functional MyD88 signaling in CD11c^+^, but not LysM^+^ cells is sufficient to induce a protective ILC3 response upon infection with *C*. *rodentium*

**Fig 1:**
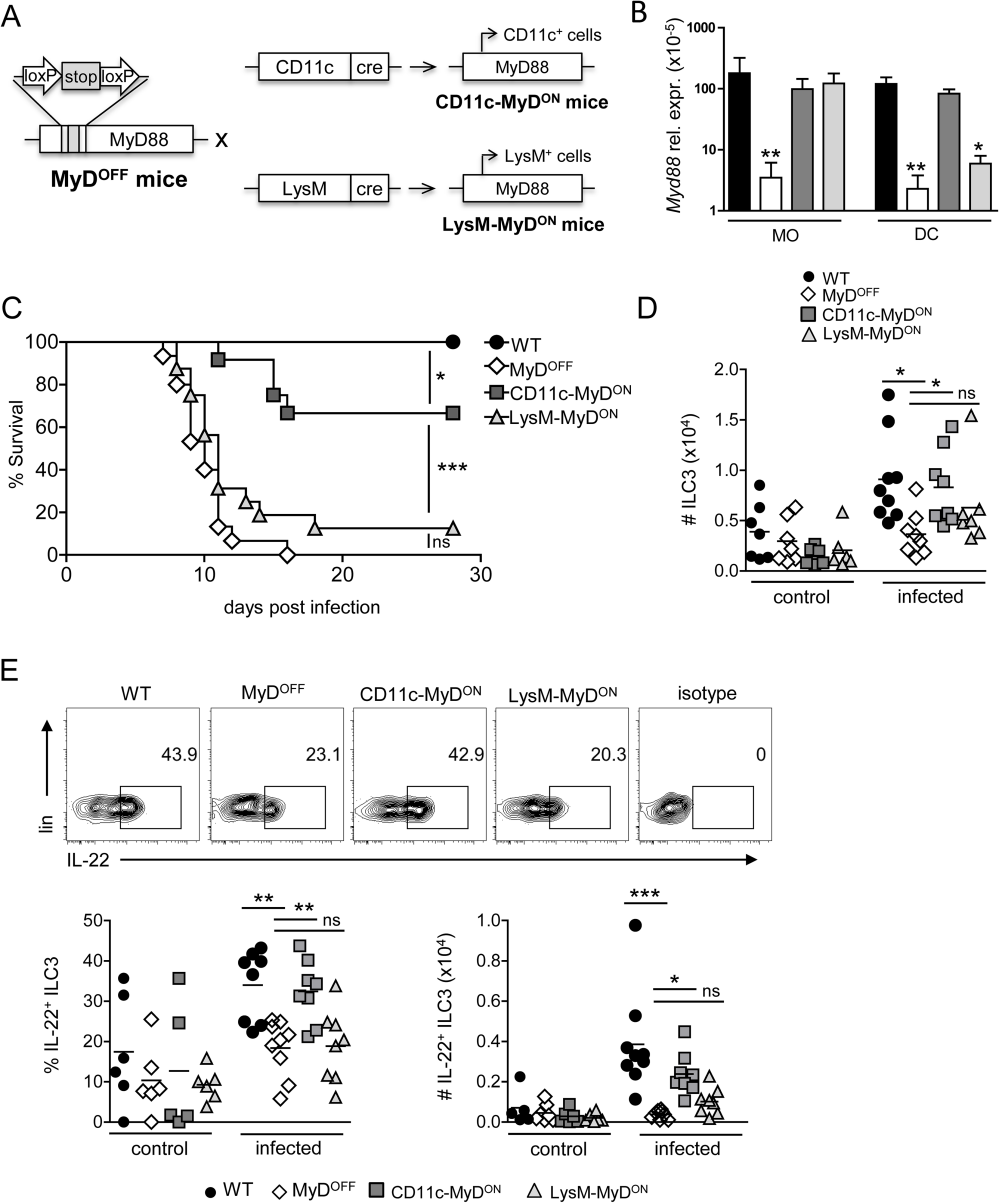
Restoring functional MyD88 signaling in CD11c^+^, but not LysM^+^ cells protects mice during infection with *C*. *rodentium*. (A) CD11c-MyD^ON^ and LysM-MyD^ON^ mice were generated by crossing MyD^OFF^ mice with CD11c-Cre or LysM-Cre mice, respectively. (B, C) WT, MyD^OFF^, CD11c-MyD^ON^ and LysM-MyD^ON^ mice were infected orally with *C*. *rodentium*. (B) On day 4 p.i., MO and DC were sorted from the cLP as live, CD64^+^F4/80^+^ (MO) or live, CD64^−^MHC-II^+^CD26^+^CD11c^+^ (DC) and tested for the expression of *Myd88* by RT-PCR. Gene expression is shown relative to the mean expression of 5 housekeeping genes (*Actb*, *B2m*, *Gapdh*, *Gusb*, *Hsp90ab1*). (C) Survival curve (D, E), leukocytes were isolated from the cLP of mice before (control) and on day 4 p.i. (infected) with *C*. *rodentium* and analyzed by flow cytometry. (D) Data depicted for the total number of lin^−^CD90^+^RORγt^+^ ILC3 within live cells (As lineage marker, antibodies against TCRβ, TCRγδ, CD19, Gr-1, Ter119, NK1.1, CD11c and CD11b were included). (E) Representative flow cytometry plots showing the frequency of IL-22^+^ cells within live ILC3 (upper panel). Graphs represent frequency (%) and total number (#) of IL-22^+^ cells amongst live ILC3 (lower panel). Data were pooled from 3 independent experiments with n = 4–5 mice (B) or n = 2–5 mice (D, E) per group. Data for the survival curve (C) were pooled from three individual experiments with a total of n = 15–20 mice per experimental group. Horizontal bar represents mean. Error bar represents +SEM. Log-rank test (C) and One-Way ANOVA with Bonferroni’s Multiple Comparison test (B, D, E); *p<0.05, **p<0.01, ***p<0.001, ns–not significant.

The key role of MyD88 for the induction of protective host responses upon infection with *C*. *rodentium* has been well established using MyD88-deficient mice, which, in contrast to wild type (WT) mice, succumb rapidly to infections with this pathogen. In order to assess the cell type-specific impact of MyD88 signaling on the induction of innate and adaptive immune responses after infection, we used a genetic approach that allows the expression of functional MyD88 in Cre-expressing MNP subsets. To this end, mice harboring a loxP-flanked stop cassette in the open reading frame of the MyD88 gene locus, disrupting functional MyD88 expression (MyD^OFF^ mice)[31], were crossed to CD11c-Cre [32] or LysM-Cre mice [33], to allow functional MyD88 expression exclusively in CD11c (CD11c-MyD^ON^ mice) or LysM (LysM-MyD^ON^ mice) positive cells, respectively (Fig 1A).

We first confirmed functional reactivation of the MyD88 locus in colonic MNP by assessing the RNA expression levels of MyD88 in DC (CD64^−^MHC-II^+^CD26^+^CD11c^+^) and MO (CD64^+^F4/80^+^MHC-II^+^), sorted from the colonic lamina propria (cLP) of the different transgenic mouse lines on day 4 after oral infection with *C*. *rodentium*. While *Myd88* expression was at the detection limit in DC and MO derived from the colon of MyD^OFF^ mice, it was expressed at WT levels in MO from both CD11c-MyD^ON^ and LysM-MyD^ON^ mice (Fig 1B). In contrast, *Myd88* expression was rescued to WT levels in DC from CD11c-MyD^ON^ mice, but was only expressed at low levels in DC derived from LysM-MyD^ON^ mice. This was in agreement with Cre-mediated RFP reporter fate mapping analysis (S1A Fig) employed to further investigate the specificity and the efficiency of the CD11c- and LysM-driven targeting of intestinal DC and MO. Using this approach, we found that CD11c-driven Cre expression efficiently targets the majority of colonic DC, with both cDC1 and cDC2 subpopulations being affected at equal frequencies (∼80%) (S1B Fig). However, we also detected targeting of colonic MO (60–70%), consistent with the expression of CD11c by MO in peripheral tissues, such as the intestine. Analysis of LysM-driven expression of RFP indicated a high targeting efficiency in colonic MHC-II^+^CD64^+^F4/80^+^ MO (∼80%), while colonic DC were affected only at low frequencies (∼5% of cDC1 and ∼ 35% of cDC2). Thus, while CD11c-driven Cre expression results in efficient targeting of both colonic DC and MO, LysM-mediated Cre expression is largely restricted to MO in the LP of the colon.

When infected orally with *C*. *rodentium*, mice with a complete deficiency in MyD88 (MyD^OFF^ mice) quickly succumbed to infection as expected (Fig 1C). Interestingly, LysM-MyD^ON^ mice were also highly susceptible and, similarly to MyD^OFF^ mice, the majority of the mice (90%) succumbed during the course of infection. In contrast, the majority of CD11c-MyD^ON^ mice initially survived the infection, although still around 40% of the animals succumbed to the infection at later stages, together indicating that functional MyD88 signaling in CD11c^+^, but not LysM^+^ cells is sufficient to induce protection during the early and also partially during the later stages of infection.

As ILC3 and ILC3-derived cytokines are known to have a prominent role in host protection during the early innate phase of infection [1–3, 34, 35], we next assessed the impact of CD11c/LysM-directed MyD88 reactivation on the ILC3 response. We observed a slight increase in the numbers of ILC3, defined as lin^−^CD90^+^RORγt^+^ cells (see S2 Fig for gating strategy), in the colon of WT and CD11c-MyD^ON^ mice 4 days after infection with *C*. *rodentium*, while ILC3 numbers in MyD^OFF^ and LysM-MyD^ON^ mice remained on a lower level (Fig 1D). Moreover, infection with *C*. *rodentium* induced significant higher frequencies of IL-22- and IL-17-secreting ILC3 in both WT and CD11c-MyD^ON^ mice as compared to MyD^OFF^ or LysM-MyD^ON^ mice (Figs 1E and S3). This was also reflected by significantly elevated total numbers of IL-22-producing ILC3 exclusively in infected WT and CD11c-MyD^ON^ mice (Fig 1E). MyD88 signaling in CD11c^+^ cells therefore may affect both recruitment and activation of ILC3 in response to the infection. Together, these data suggest that MyD88-mediated signaling in CD11c^+^ MNP, but not MO alone, is critical and sufficient to activate the colonic ILC3 response, and to efficiently protect mice during the early phase of infection with *C*. *rodentium*.

### MyD88 signaling in CD11c^+^ cells restores the colonic inflammatory response

**Fig 2:**
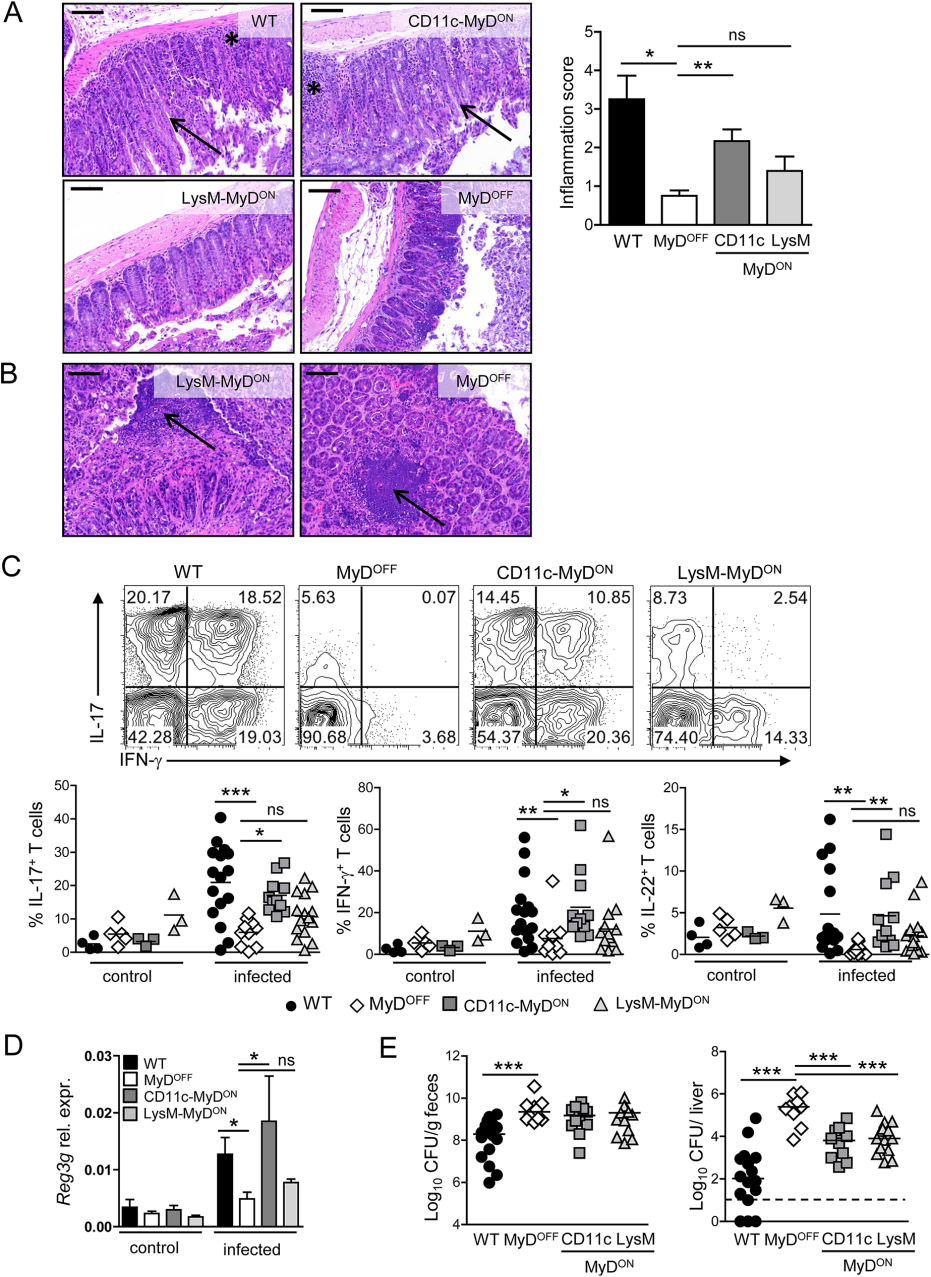
MyD88 signaling in CD11c^+^ cells is sufficient to induce an inflammatory response in the colon of mice infected with *C*. *rodentium*. (A, B) Representative H&E staining of colon sections of WT, MyD^OFF^, CD11c-MyD^ON^ and LysM-MyD^ON^ mice on day 8–10 p.i. with *C*. *rodentium*. Black asterisks indicate inflammatory infiltration into the cLP; black arrow heads indicate crypt elongation (A) or gangrenous-like necrotic structures (B). Scale bar represents 100 μm. Bar graph shows inflammation score based on infiltration and epithelial hyperplasia. (C) Leukocytes were isolated from the cLP of mice before (control) or on day 8 p.i. (infected) with *C*. *rodentium* and the T cell response was analyzed by flow cytometry. Representative flow cytometry plots of live CD3^+^CD4^+^ T cells stained for IL-17A and IFN-γ (upper panel). Graphs represent frequency (%) of IL-17A^+^ (IFN-γ^+/−^), IFN-γ^+^ (IL-17^+/−^) and IL-22^+^ cells amongst live CD3^+^CD4^+^ T cells (lower panel). (D) Expression of *Reg3g* in colonic epithelial cells isolated from mice before (control) or on day 8 p.i. (infected) with *C*. *rodentium*. Gene expression is shown relative to *Actb*. (E) Bacterial load in the feces and the liver on day 8–10 p.i. Data were pooled from two (A) or four (C, D) independent experiments with n = 3 mice (A) or n = 1–4 mice (C, D) per group. Horizontal bar represents mean. Error bar represents +SEM. Dashed line indicates the limit of detection. One-Way ANOVA with Bonferroni’s Multiple Comparison test; *p<0.05, **p<0.01, ***p<0.001, ns–not significant.

While the colon of uninfected mice from all genotypes had a normal healthy appearance (S4 Fig), the activation of host defense mechanisms in *C*. *rodentium* infected WT mice induced colonic pathology characterized by epithelial cell hyperplasia, which leads to crypt elongation, as well as a pronounced inflammatory cellular infiltration into the cLP (Fig 2A, black arrows and asterisks). In infected MyD^OFF^ mice however, these typical signs of an inflammatory pathology were absent (Fig 2A). Consistently with what has been reported previously for MyD88^−/−^ mice [25, 28], colonic tissue from MyD^OFF^ mice instead displayed distinct necrotic epithelial injury and gangrenous mucosal damage associated with bacterial microcolonies (Fig 2B, black arrows). Similar pathologic features were present in the colon of LysM-MyD^ON^ mice (Fig 2B), although we detected a slight increase in the inflammatory cellular infiltration into the cLP in comparison to MyD^OFF^ mice. In contrast, no gangrenous mucosal damage was detected in CD11c-MyD^ON^ mice. Instead, crypt elongation and cellular infiltration into the cLP was largely restored in these mice (Fig 2A, black arrows and asterisks), indicating that reactivation of MyD88 in CD11c^+^ cells governs functional induction of inflammatory host defense mechanisms in the gut. In line with these findings, the amount of infiltrating neutrophils was increased in the colon of WT and CD11c-MyD^ON^ mice, and we observed a trend toward enhanced numbers of MO in the cLP of WT, CD11c-MyD^ON^ and LysM-MyD^ON^ mice, as compared to MyD^OFF^ mice (S5 Fig). In contrast, the frequencies and total numbers of DC were similar in the colon of mice from all genotypes (S5 Fig).

Besides the activation of ILC, protective immunity to *C*. *rodentium* infection requires in addition the induction of a potent T cell response. We therefore next analyzed the impact of specific MyD88 reactivation on the colonic T cell response on day 8–10 p.i. Interestingly, we observed an almost complete lack of IL-17A-, IFN-γ- or IL-22-secretion by T cells isolated from the cLP of MyD^OFF^ mice (Fig 2C). While colonic T cells from LysM-MyD^ON^ mice showed mild, albeit non-significant higher frequency and number of cytokine-producing T cells, restoration of MyD88 signaling in CD11c^+^ cells resulted in a significantly elevated frequency and total number of IL-17A-, IFN-γ- and IL-22-producing CD4^+^ T cells in the colon (Figs 2C and S6). In accordance with the induction of a substantial amount of IL-22/IL-17 -producing cells in CD11c-MyD^ON^ mice, we found that the expression of the antimicrobial peptide RegIIIγ from the colonic epithelium was also restored to WT levels in those mice (Fig 2D). Despite these findings, the bacterial burdens measured on day 8–10 p.i. in the feces and the liver of CD11c-MyD^ON^ mice were not reduced to the level detected in WT mice, although there was a clear reduction in liver CFU as compared to MyD^OFF^ mice (Fig 2E). In line with the results of the survival experiments shown in Fig 1C, these data suggest that the re-establishment of host defense mechanisms in CD11c-MyD^ON^ does not suffice to induce complete protection from infection. Of note, CFU were also reduced in the liver of LysM-MyD^ON^ mice, which may indicate that although these mice cannot control the infection, the slight induction of the inflammatory T cell response still has a measurable effect on the systemic bacterial burden.

### Restoring MyD88 signaling in CD11c^+^ cells triggers activation of colonic DC in *C*. *rodentium* infected mice

**Fig 3:**
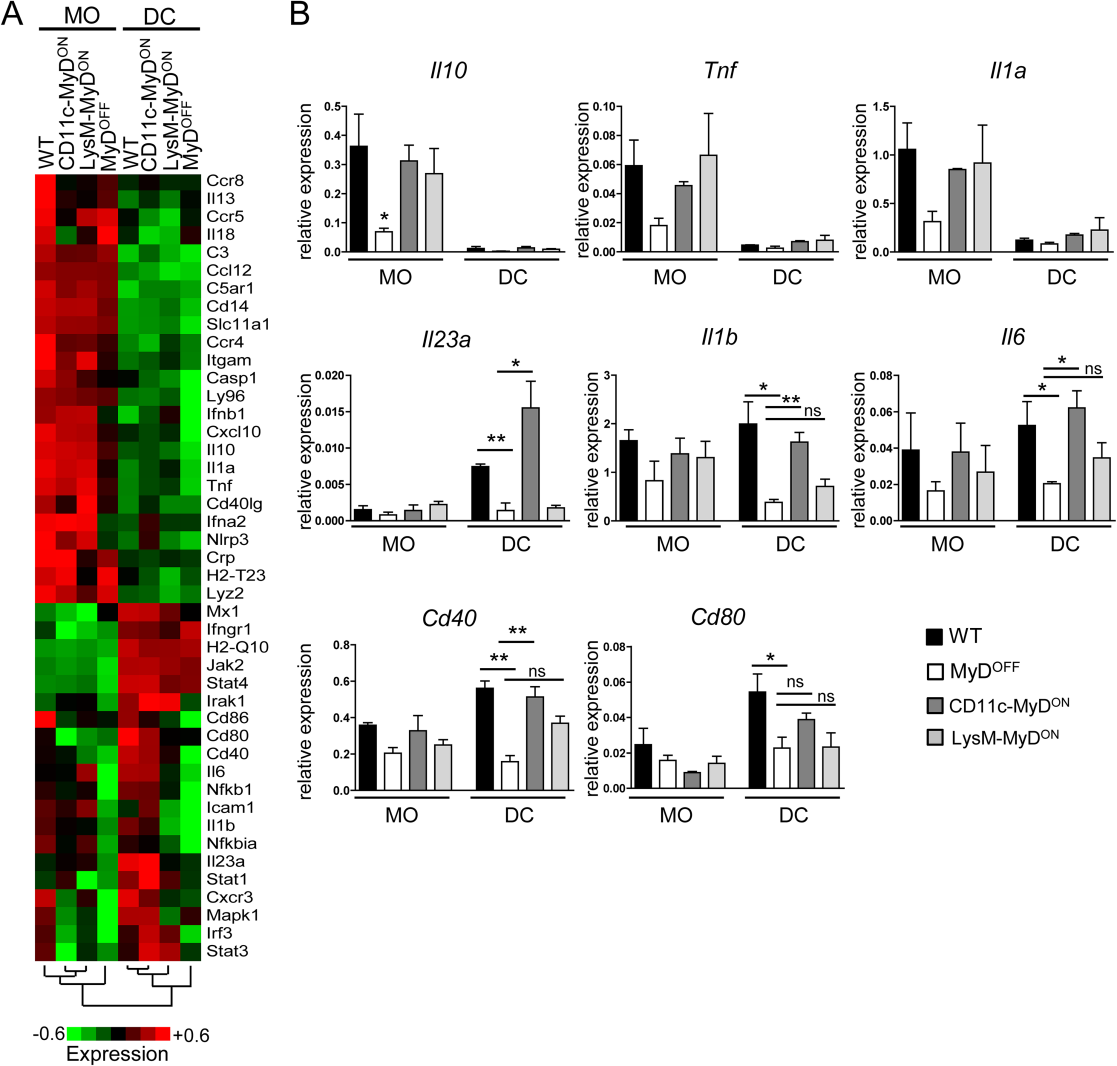
Impact of MyD88 reactivation on the expression of factors that govern MO/DC function in infection and inflammation. (A, B) WT, MyD^OFF^, CD11c-MyD^ON^ and LysM-MyD^ON^ mice were infected orally with *C*. *rodentium*. On day 4 p.i. leukocytes were isolated from the cLP and sorted for DC (lin^−^CD64^−^MHC-II^+^CD26^+^CD11c^+^) and MO (lin^−^CD64^+^F4/80^+^MHC-II^+^). As lineage marker, antibodies against CD3, CD19, B220 and NK1.1 were included (A) Heat map displaying expression of genes involved in MNP function in infection and inflammation analyzed by real-time PCR from sorted DC/MO fractions. Data presented as mean log2 value of relative mRNA expression (see color scale). (B) Bar graphs show mean expression of selected genes in sorted DC/MO fractions relative to the expression of 5 housekeeping genes (*Actb*, *B2m*, *Gapdh*, *Gusb*, *Hsp90ab1*). Data were pooled from three individual experiments with n = 4–6 mice per group. Error bar represents +SEM. One-Way ANOVA with Bonferroni’s Multiple Comparison test; *p<0.05, **p<0.01, ns–not significant.

In order to determine factors that are activated in DC and MO after specific MyD88 reactivation in CD11c-MyD^ON^ and LysM-MyD^ON^ mice, we next sorted DC and MO from the cLP of infected mice (S7 Fig) and assessed the transcriptional level of genes associated with MNP activation and function in infection and inflammation. Cluster analysis revealed a number of genes highly expressed in MO from all genotypes, such as *Lyz2* (LysM) or *Itgam* (Mac-1), but with low expression in DC, together confirming that our sorting approach faithfully discriminated colonic MO from DC (Fig 3A). Among the factors highly expressed in MO were also *Il10*, *Tnf* and *Il1a*, which however showed low expression in MO derived from MyD^OFF^ mice (Fig 3B), indicating that MO-specific MyD88 signaling is critical to upregulate or sustain transcription of these genes, respectively. We also identified a set of genes that displayed increased expression levels in DC isolated from WT and CD11c-MyD^ON^ mice as compared to DC from LysM-MyD^ON^ and MyD^OFF^ mice (Fig 3A and 3B). Most prominent among those was *Il23a*, which was highly expressed only in WT and CD11c-MyD^ON^-derived DC, but not in DC and MO from all other genotypes (Fig 3B). In addition, we found that the expression of *Il1b* and *Il6* was restored also in DC derived from CD11c-MyD^ON^, but not LysM-MyD^ON^ mice to levels detected in WT mice (Fig 3B). Together, these findings indicate that restricting functional MyD88 signaling to CD11c^+^, but not LysM^+^ cells is critical and sufficient for the expression of cytokines by DC that play a key role in ILC3 activation and Th17 induction. Of note, we also observed a trend toward increased transcript expression of costimulatory molecules such as CD40 and CD80 in DC from CD11c-MyD^ON^ mice, together indicating that CD11c-restriced MyD88 signaling is sufficient to induce the whole spectrum of DC activation after infection with *C*. *rodentium*.

### Restoring MyD88 signaling in T cells and ILC3 is not sufficient to induce host immune mechanisms upon intestinal infection

**Fig 4:**
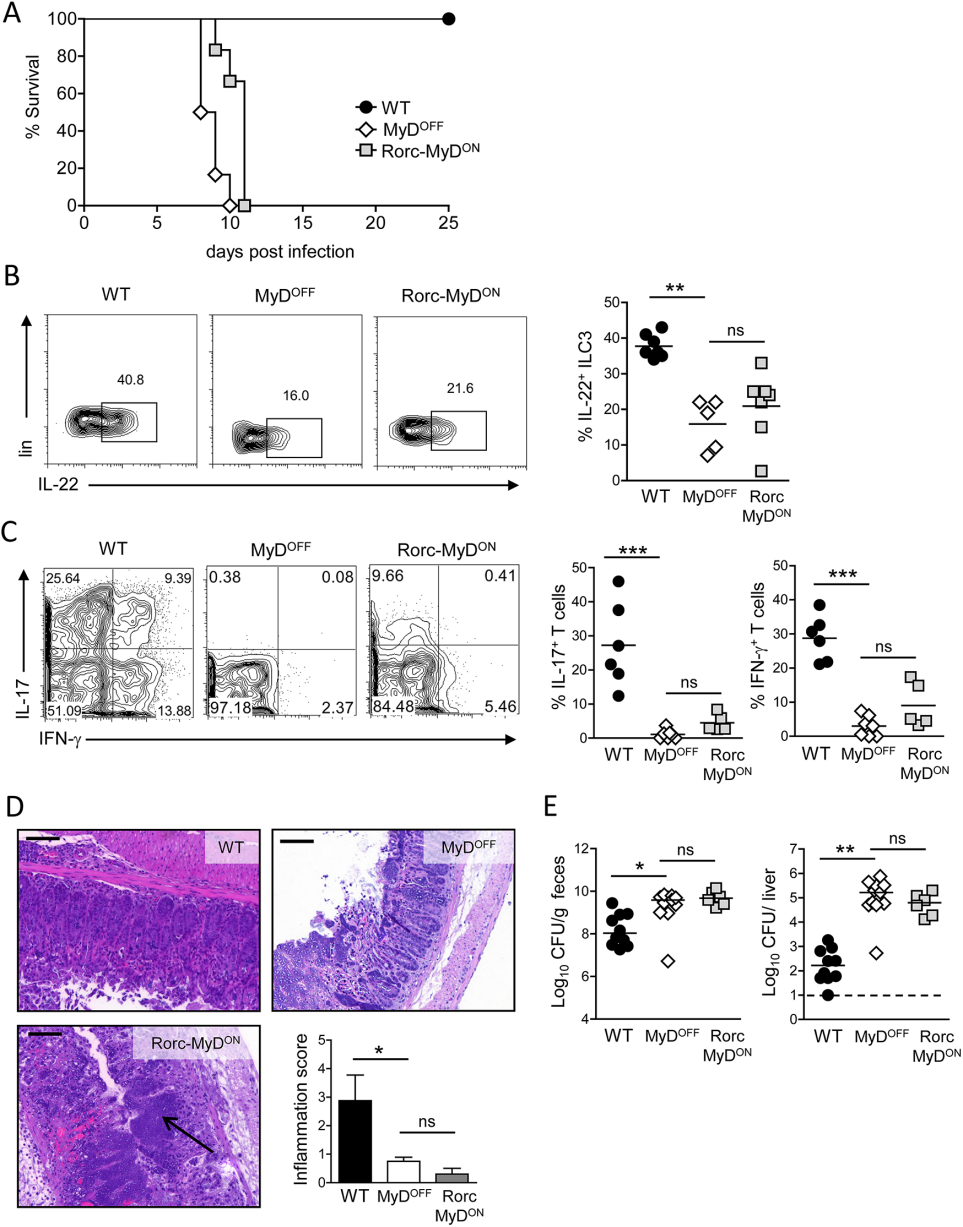
MyD88 signaling in T cells and ILC3 is not sufficient to induce host immune responses. (A) WT, MyD^OFF^ and Rorc-MyD^ON^ mice were infected orally with *C*. *rodentium* and monitored for survival. (B) Representative flow cytometry plots showing the frequency of colonic IL-22^+^ cells within live ILC3 on day 4 p.i. Graphs represent frequency (%) of IL-22^+^ cells amongst live ILC3. (C) Colonic T cell response on day 8 p.i. Representative flow cytometry plots of live CD3^+^CD4^+^ T isolated from the cLP and stained for IL-17A and IFN-γ. Graphs represent frequency (%) of colonic IL-17A^+^ (IFN-γ^+/^) and IFN-γ^+^ (IL-17^+/−^) cells amongst live CD3^+^CD4^+^ T cells. (D) Representative H&E staining of colon sections of WT, MyD^OFF^ and Rorc-MyD^ON^ mice on day 8 p.i. with *C*. *rodentium*. Scale bar represents 100 μm. Black arrow head indicates distinct necrotic epithelial injury and gangrenous mucosal damage in the colon of Rorc-MyD^ON^ mice. Bar graph shows inflammation score based on infiltration and epithelial hyperplasia. (E) Bacterial load in the feces and the liver on day 8 p.i. Data are representative for two independent experiments with n = 6 mice per group (A) or were pooled from two (B-D) or three (E) independent experiments with n = 2–5 mice per group. Horizontal bar represents mean. Error bar represents +SEM. Dashed line indicates the limit of detection. One-Way ANOVA with Bonferroni’s Multiple Comparison test; *p<0.05, **p<0.01, ***p<0.001, ns–not significant.

Recent studies have shown that TLR-MyD88-dependent signaling in CD4^+^ T cells is required to coordinate intestinal IgA responses and therefore critically controls normal gut homeostasis [36, 37]. In addition, it has been reported that T cell-specific TLR2 activation can promote Th17 responses [38]. Besides this direct TLR-mediated activation, IL-1R-MyD88-mediated signaling in T cells plays an important role for the induction of Th17 responses [39, 40], which is consistent with the prominent role of IL-1β in the microbiota-induced development of Th17 cells in the intestine [41]. Murine ILC do not express TLR, however, IL-1β has been suggested to be a potent driver of intestinal ILC3 function via activation of IL-1R-MyD88-mediated signaling in those cells [42]. In order to investigate the influence of MyD88 signaling specifically in T cells and ILC3 during intestinal infection, we generated Rorc-MyD^ON^ mice by crossing MyD^OFF^ mice with mice expressing Cre under the promotor of *Rorc(γt)* [43]. Since RORγt is highly expressed in thymocytes at the double positive stage, MyD88 is reactivated in ILC3 and in all T cells in Rorc-MyD^ON^ mice. When infected orally with *C*. *rodentium*, Rorc-MyD^ON^ mice rapidly succumbed to the infection with similar kinetics to MyD^OFF^ mice (Fig 4A). Moreover, reactivation of MyD88 signaling in RORγt^+^ cells neither restored IL-22 production by colonic ILC3 (Fig 4B), nor led to the induction of an intestinal Th1/Th17 cell response in Rorc-MyD^ON^ mice (Fig 4C). In line with these results, the colonic pathology in Rorc-MyD^ON^ mice was characterized by epithelial injury and gangrenous mucosal damage without the establishment of inflammatory type pathologic changes in the colon (Fig 4D). Furthermore, we observed high bacterial loads in the feces and the liver of Rorc-MyD^ON^ mice (Fig 4E), overall confirming that ILC3- and T cell-restricted functional MyD88 expression is not sufficient to induce an appropriate host response to infection with *C*. *rodentium*.

Notably, expression of CD11c has been reported in a fraction of activated intestinal T cells [44, 45]. Using CD11c-Cre mediated RFP fate mapping, we in fact observed that ∼25% of colonic CD4^+^ T cells, as well as a minor fraction of ILC3 (∼10%) were RFP-labelled upon infection with *C*. *rodentium* (S8A and S8B Fig). However, the inability of both Rorc-MyD^ON^ and LysM-MyD^ON^ mice to activate substantial host response mechanisms upon infection clearly suggests that the pronounced establishment of the immune response that we observed in CD11c-MyD^ON^ mice was not a consequence of functional MyD88 expression in T cells, ILC3 or MO, but indeed due to functional MyD88 signaling in DC.

### Specific reactivation of functional MyD88 signaling in the intestinal epithelium enhances host resistance to infection

**Fig 5:**
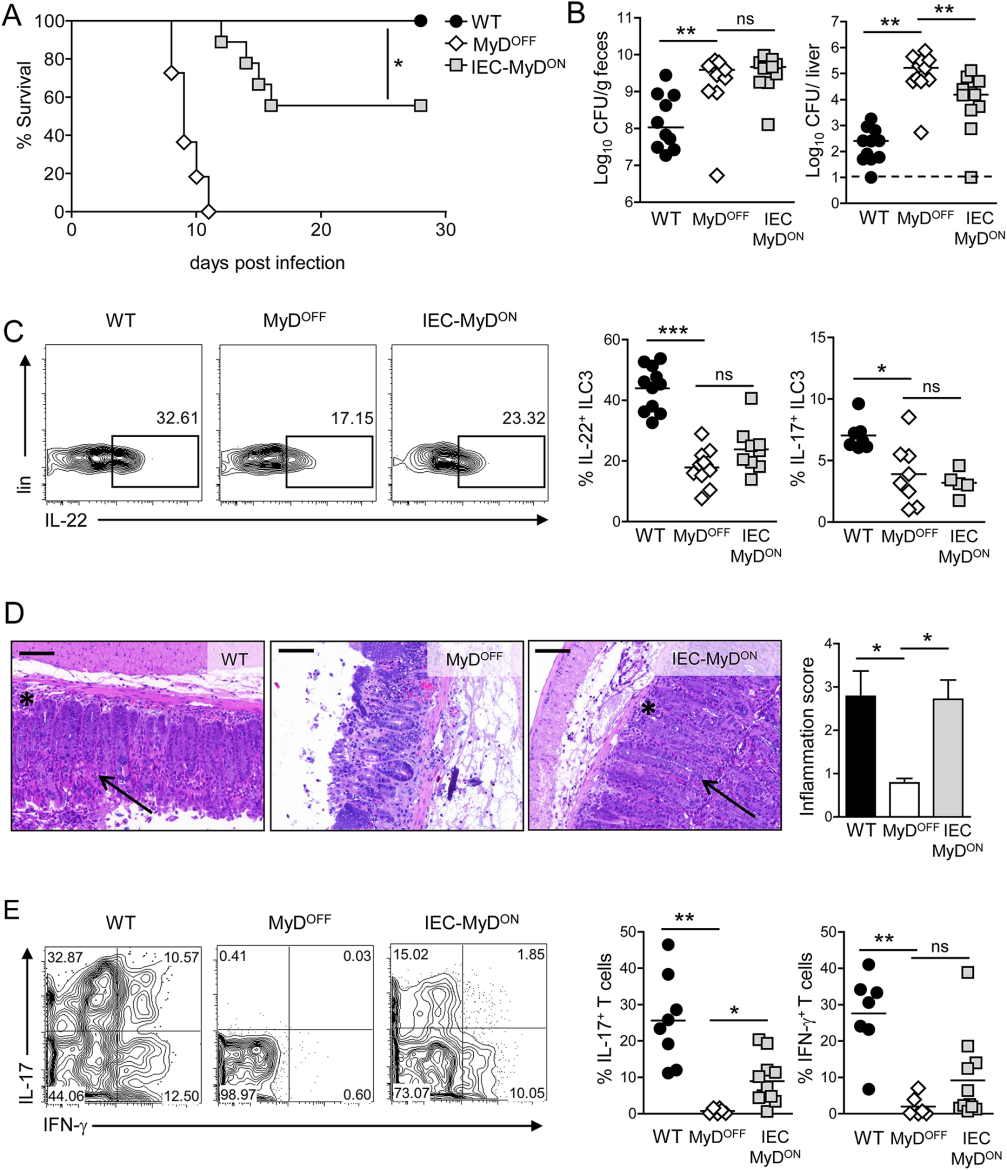
MyD88-dependent signaling in IEC contributes to host resistance to infection. (A) Survival of WT, MyD^OFF^ and IEC-MyD^ON^ mice following infection with *C*. *rodentium*. (B) Bacterial load in the feces and the liver on day 8 p.i. (C) ILC3 response on day 4 p.i. with *C*. *rodentium*. Cells were isolated from the cLP and analyzed by flow cytometry. Representative plots showing the frequency of IL-22^+^ cells within live ILC3. Graphs represent frequency (%) of IL-22^+^ and IL-17A^+^ cells amongst live ILC3. (D) Representative H&E colon sections on day 8 p.i. with *C*. *rodentium*. Black asterisks indicate inflammatory infiltration into to the cLP; black arrow heads indicate crypt elongation. Scale represents 100 μm. Bar graph represents inflammation score based on infiltration and epithelial hyperplasia. (E) Colonic T cell response on day 8 p.i. Representative flow cytometry plots of live CD3^+^CD4^+^ T isolated from the cLP and stained for IL-17A and IFN-γ. Dot plots represent frequency (%) of colonic IL-17A^+^ (IFN-γ^+/−^) and IFN-γ^+^ (IL-17^+/−^) cells amongst live CD3^+^CD4^+^ T cells. Data were pooled from two individual experiments with a total of n = 11 mice per group (A) or pooled from two (D) or three (B, C, E) independent experiments with n = 2–5 mice per group. Horizontal bar represents mean. Error bar represents +SEM. Dashed line indicates the limit of detection. Log-rank test (A) and One-Way ANOVA with Bonferroni’s Multiple Comparison test (B-E); *p<0.05, **p<0.01, ***p<0.001, ns–not significant.

Our data so far have demonstrated that MyD88-dependent signaling in CD11c^+^ cells is sufficient to activate DC and the subsequent colonic ILC3 and T cell response. However, our experiments also revealed that CD11c-MyD^ON^ mice did not completely mirror WT mice in terms of survival and bacterial clearance, indicating that MyD88-dependent signaling is still required in additional cell types for full protection. It is well established that IEC express a broad range of TLR, and that triggering of TLR-mediated pathways in these cells substantially influences proliferation, homeostasis and repair mechanisms of the epithelium [46]. In order to investigate the consequences of IEC-specific MyD88 activation on the host defense upon intestinal infection, we generated IEC-MyD^ON^ mice by crossing MyD^OFF^ mice with mice expressing Cre under the promotor of *Villin* [47], thus allowing the efficient expression of MyD88 in IEC (S9 Fig). In contrast to what we observed in MyD^OFF^ mice, animals with an IEC-specific MyD88 reactivation survived the early phase of infection with *C*. *rodentium*. However, as described above for CD11c-MyD^ON^ mice, around 50% of the IEC-MyD^ON^ mice succumbed to the infection at a later stage (Fig 5A). In line with this, we observed a partial reduction of the bacterial load in the liver, but not the feces of IEC-MyD^ON^ mice as compared to MyD^OFF^ mice (Fig 5B). Interestingly, IL-22/IL-17 production by ILC3 isolated from the cLP of IEC-MyD^ON^ mice remained at similar basal levels to that observed in MyD^OFF^ mice (Fig 5C), indicating that the increased resistance of IEC-MyD^ON^ mice to infection is not a consequence of restored ILC3 function. In contrast, the histopathologic analysis of colons derived from IEC-MyD^ON^ mice revealed the typical characteristics of infection-induced inflammation seen in WT mice, including crypt elongation and inflammatory cellular infiltration (Fig 5D). This was also associated with a modest increase in the frequencies of IL-17- and IFN-γ-producing T cells in the colon of IEC-MyD^ON^ mice (Fig 5E), together suggesting that IEC-restricted functional MyD88 expression, although it does not rescue the local ILC3 response upon infection, still allows for a partial induction of inflammatory host responses in the colon.

### MyD88 signaling in IEC enhances the intestinal barrier function

**Fig 6:**
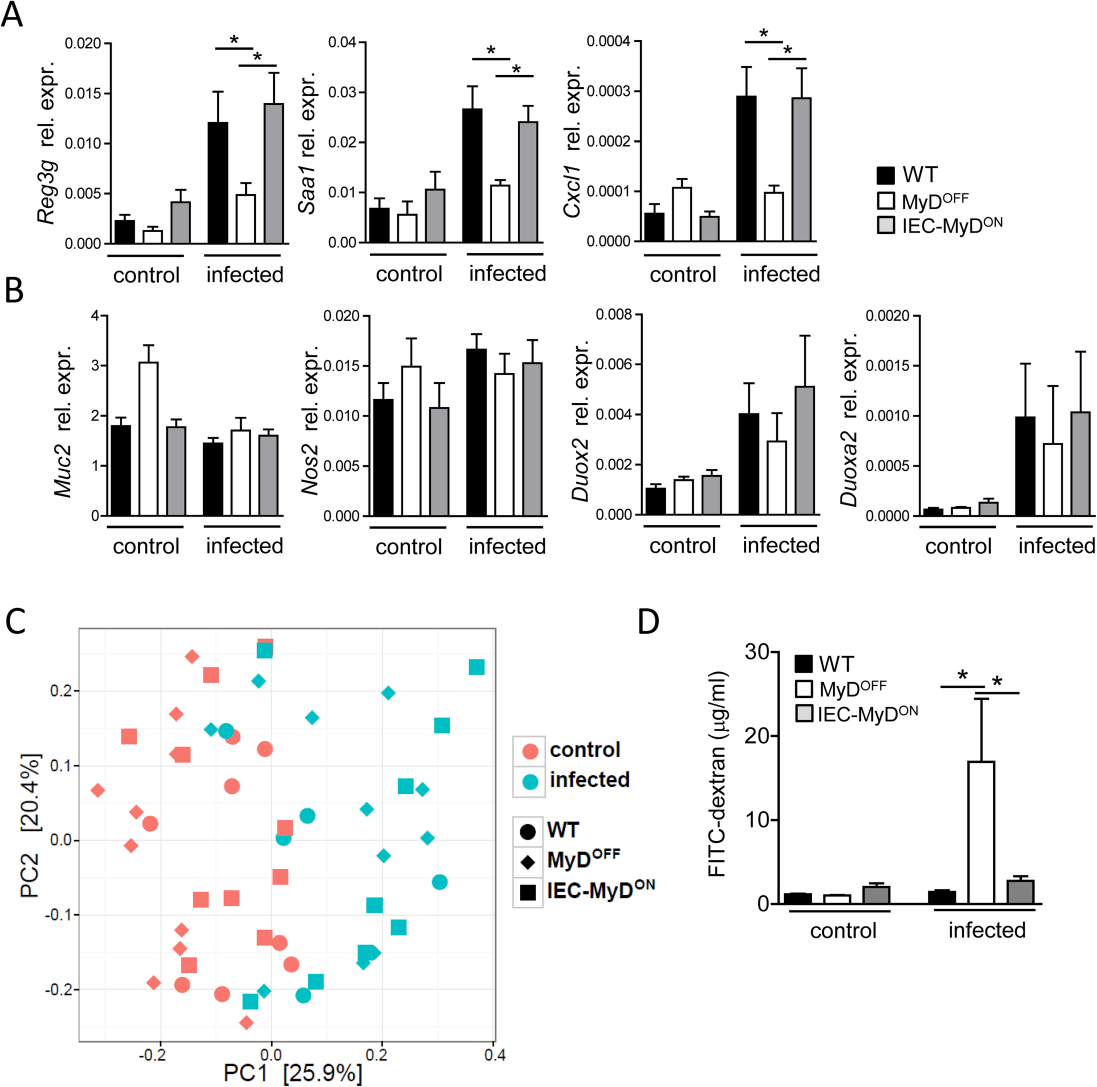
IEC intrinsic MyD88 signaling promotes barrier function of the epithelium. (A, B) Gene expression in IEC isolated before (control) or on day 4 p.i. (infected) with *C*. *rodentium* from the colon of WT, MyD^OFF^ and IEC-MyD^ON^ mice. Data shown as mean relative expression to *Actb*. (C) Principal component analysis of the intestinal microbiota in individual mice before (control) or on day 4 p.i. (infected) with *C*. *rodentium*. (D) Intestinal permeability in WT, MyD^OFF^ and IEC-MyD^ON^ mice before (control) or on day 8 p.i. (infected) with *C*. *rodentium*. FITC-dextran serum levels were determined 4 h after oral administration of this compound. Data were pooled from two (C), three (D) or four (A, B) independent experiments with n = 3–5 mice per group. Error bar represents +SEM. One-Way ANOVA with Bonferroni’s Multiple Comparison test (A, B, D); *p<0.05, **p<0.01, ***p<0.001.

To further elucidate the mechanism by which IEC-restricted MyD88 signaling contributes to enhanced protection from intestinal infection, we analyzed the IEC-specific expression of factors involved in host resistance to Citrobacter. We observed that IEC-specific expression of MyD88 signaling in IEC-MyD^ON^ mice was sufficient to upregulate the transcription of the antimicrobial peptide RegIIIγ in IEC (Fig 6A). In addition, transcription of the acute-phase protein SAA1 as well as of the neutrophil-recruiting chemokine CXCL1 was upregulated in MyD88-proficient IEC, which together may contribute to the increased inflammatory pathology that we observed in the colon of IEC-MyD^ON^ mice (Fig 5D). While these findings suggest that IEC-intrinsic MyD88 activation is sufficient to trigger the expression of several important epithelium-derived factors, we found that the expression of other molecules implicated in epithelial defense upon intestinal infection with *Citrobacter*, such as inducible nitric oxide synthase 2 (Nos2), reactive oxygen species (ROS)-generating enzyme dual oxidase 2 (Duox2) and its maturation factor Duoxa2 [48], was independent of MyD88 signaling in IEC (Fig 6B). Although it is described as being MyD88-dependent, we also did not find differences in the expression of *Muc2*, which encodes for the primary mucin component of the mucus layer and plays an important role for host defense after infection with *C*. *rodentium* [49, 50].

Recent studies have suggested that MyD88-deficiency may affect the composition of the colonic microbiota [51, 52]. Thus, IEC-specific expression of MyD88 may influence the composition of the microbiota and, as a consequence, the susceptibility toward *C*. *rodentium* infection. In order to test this hypothesis, we assessed the fecal microbiome of our experimental mice by 16S rRNA gene sequencing. Principal component analysis revealed that the composition of the microbiota was significantly changed upon infection according to permutational MANOVA test (p<0.001) **(**Fig 6C). A detailed analysis of the microbiota on the family level showed that the difference observed between pre- and post-infection was largely due to the strong expansion of the family *Enterobacteriaceae*, most likely reflecting the colonization with *C*. *rodentium* (S10 Fig). However, functional expression of MyD88 in IEC did not result in significant changes in the composition of the microbiota as compared to MyD^OFF^ mice (permutational MANOVA, p>0.5), suggesting that the difference in the susceptibility to infection observed between these genotypes is not a consequence of an altered microbiota composition.

We finally tested whether restricting functional expression of MyD88 to IEC has a direct impact on the epithelial barrier integrity during infection. To this end we measured serum levels of FITC-dextran 4 hours after oral gavage of this compound, as a read-out for intestinal epithelial permeability. Although we did not observe differences in epithelial permeability under steady-state conditions, infection with *C*. *rodentium* resulted in a strong increase in epithelial permeability in MyD^OFF^ mice as compared to WT controls, suggesting that loss of MyD88 function severely impairs the epithelial barrier integrity during infection (Fig 6D). IEC-restricted MyD88 expression in IEC-MyD^ON^ mice however led to a significant decrease in FITC-dextran serum levels, revealing an important IEC-intrinsic role of MyD88 in controlling epithelial integrity upon infection with *C*. *rodentium*. Taken together, our data highlight the critical importance of MyD88-dependent signaling in CD11c^+^ DC and IEC for protection against intestinal bacterial infection. While DC are protective due to their induction of strong local ILC3 and T cell responses, MyD88 signaling in IEC seems to mainly affect the barrier function of the epithelium.

## Discussion

Infection with *C*. *rodentium* triggers innate and adaptive inflammatory immune responses in the intestine. This pathogen therefore represents an ideal model to study the contribution of innate cells such as ILC to intestinal defense mechanisms, including the regulation of epithelial barrier function, and the processes leading to T cell-mediated intestinal inflammation. The pivotal role of the adapter protein MyD88 in TLR-mediated activation of innate immunity upon recognition of microbe associated molecular patterns (MAMP) has been well established. In addition, MyD88 mediates signal transduction by the IL-1 receptor family, thereby contributing to TLR-independent activation of innate and adaptive immune cells in a cell-intrinsic manner. Although MyD88-mediated pathways are in general considered to be proinflammatory, several studies have also found a critical role for MyD88 in protecting from colitis by promoting intestinal homeostasis [53, 54]. Moreover, MyD88 signaling in both hematopoietic as well as non-hematopoietic cells and tissues has been suggested to contribute to host defense and mucosal tissue protection following intestinal infection and inflammation [17, 28, 50, 51, 55–58]. However, despite the key role of MyD88 in governing intestinal immunity, a thorough understanding of the cell type-specific impact of MyD88 in the activation of the different innate and adaptive intestinal immune responses upon infection has been lacking so far.

In the present study, we determined the role of MyD88 in several cell types using a mouse model for Cre-mediated activation of functional MyD88. Importantly, this gain-of-function model restricts functional MyD88 signaling to a given Cre-expressing cell or tissue type, while all other cells of the body remain deficient for MyD88. We and others have shown that this recently developed MyD^ON^ model allows the precise study of direct consequences of cell type-specific TLR/IL-1R-mediated signaling on the immune response [31, 59–62].

Previous studies highlighted the importance of the cytokine IL-22 for inducing resistance against infection with *C*. *rodentium* [1]. ILC3 have been suggested as a critical source of this cytokine during the early phase of infection [2, 3, 35], although a substantial level of redundancy between ILC and Th17/Th22 cells in *C*. *rodentium* infections has been reported recently [63]. Our data is in line with an important role for ILC3 and ILC3-derived IL-22 during the early phase of infection with *C*. *rodentium*. This may be explained by the absence of pre-existing Th17/22 cells in the intestine of the mice from our animal facility. In accordance with a report by Rynders *et al*., we observed a low level of IL-22 production by ILC3 in MyD88-deficient animals under steady-state conditions and during infection [42], indicating that basal production of IL-22 by ILC3 may largely be regulated independently from MyD88-mediated signals. However, ILC3 from MyD88-deficient animals failed to upregulate the production of IL-22 and IL-17 upon intestinal infection, suggesting that MyD88-mediated signaling is strictly required for the functional activation of ILC3 after pathogenic challenge. Moreover, reactivation of MyD88 exclusively in CD11c^+^ cells completely corrected this inability of ILC3 to upregulate IL-22 and IL-17, while reactivation of MyD88 in LysM^+^ cells had no effect on the colonic ILC3 response. Considering our finding that exclusively DC from WT and CD11c-MyD^ON^ mice, but neither MO nor DC from all other genotypes upregulated IL-23 expression, we conclude that DC- but not MO-specific MyD88 signaling is both crucial and sufficient to fully induce ILC3 activation in *C*. *rodentium* infection. This is in line with data showing that mice with a CD11c-specific deletion of MyD88 display enhanced susceptibility towards infection with *C*. *rodentium*, which could be rescued by hydrodynamic injection of a plasmid expressing IL-22 [17]. Moreover, *ex vivo* stimulation of intestine-derived CD103^+^CD11b^+^ DC with the TLR5 ligand flagellin increased the expression of IL-23 and enhanced ILC3 responses [22], further emphasizing the importance of MyD88 signaling in DC for ILC3 activation. Although it has been suggested that CX3CR1-expressing intestinal MO can promote IL-22 production by ILC3 [17, 18], the role of MO-specific MyD88 signaling in this process was not clear. Our data indicate that MyD88 expression in MO alone is not sufficient to activate an appropriate ILC3 response after infection, which is consistent with the notion that these cells are rather refractory to TLR stimulation, at least under homeostatic conditions [64].

Our data using Rorc-MyD^ON^ mice also show that re-establishing MyD88 signaling in ILC3 is not sufficient to induce activation of these cells. This may be explained by a general lack of IL-1β when MyD88 is not expressed in other cell types such as intestinal DC. Rynders *et al*. have shown that ILC3-intrinsic IL-1R/MyD88 signaling contributes to IL-22 production under homeostatic conditions [42]. However, in CD11c-MyD^ON^ mice ILC3 are activated normally, indicating that the lack of MyD88 in these cells does not critically interfere with upregulation of IL-22/IL-17.

Besides the consequences for the early activation of ILC3, we also demonstrate here that CD11c-driven MyD88 signaling is sufficient to induce colonic T cell responses with frequencies of IL-17A-, IFN-γ- and IL-22-producing T cells comparable to those in WT mice. In this regard, it has been noted that MyD88 signaling regulates migration of DC to mesenteric lymph nodes, where T cell priming occurs [65–67]. Moreover, treatment of lamina propria- or mesenteric lymph node-derived DC with TLR ligands (e.g. flagellin, LPS, CpG ODN) *in vitro*, promoted the differentiation of naïve T cells into Th17, Th22 and Th1 cells, which was attributed to the production of pro-inflammatory cytokines such as IL-6, IL-1β and IL-12 [20, 68–70]. This is in accordance with our finding of enhanced expression of both IL-6 and IL-1β in DC from WT and CD11c-MyD^ON^ mice, together, suggesting that MyD88-dependent activation of DC is indeed sufficient to create the micro-environment required to induce Teff cell differentiation upon intestinal infection. Nevertheless, we cannot exclude that beside the activation of DC function in CD11c-MyD^ON^ mice, MyD88 reactivation in intestinal MO in these mice may still contribute to the protective host responses against *C*. *rodentium*.

In contrast to the DC-specific effects, restricting MyD88-dependent signaling to LysM^+^ cells did not promote significant colonic host T cell responses. We did, however, note a minor increase in Teff cell frequencies in LysM-MyD^ON^ mice compared to constitutive MyD88-deficient MyD^OFF^ mice. It is possible that MyD88-dependent signals in MO, although not sufficient to induce initial activation and priming of naïve T cells, can contribute to Teff cell expansion at a later stage [71]. Nevertheless, we cannot rule out that additional LysM-driven targeting of other cell types such as small frequencies of intestinal CD172a^+^ cDC2 may contribute to the low level of host responses that we observed in LysM-MyD^ON^ mice. Moreover, although we showed here that Rorc-Cre-mediated MyD88-reactivation in T cells is not sufficient to induce a substantial colonic T cell response upon infection with *C*. *rodentium*, it is still possible that reactivation of MyD88 in a subset of activated T cells in CD11c-MyD^ON^ mice additionally contributes to the development of the colonic T cell response, consistent with reports demonstrating that T cell-intrinsic IL-1R/MyD88-dependent signaling promotes the induction, survival and proliferation of Th17 cells [39, 40, 72].

The data presented in this study indicate that besides MyD88 signaling in DC, IEC-specific MyD88-mediated signals also contribute to specific host defense mechanisms during Citrobacter infection. Previous studies that addressed an IEC-specific role of MyD88 using conditional MyD88 targeting in IEC have found little evidence for a prominent role of IEC-intrinsic MyD88 in colitis or infection with *C*. *rodentium* [57, 73]. More recently, it was reported that mice with an IEC-specific deletion of MyD88 display defects in Muc2 expression and crypt antimicrobial capacity in the cecum during the early phase of infection with *C*. *rodentium* [50]. Nevertheless, this defect was only transient and did not significantly affect bacterial burden or intestinal barrier permeability. In contrast to studies using mice with conditional deletion of MyD88 in IEC, here we reactivated MyD88 exclusively in IEC, which allowed us to directly follow the effects of functional MyD88 signaling in these cells in the absence of compensating effects from other MyD88-expressing cell types. We show that functional MyD88 in IEC alone is indeed sufficient to upregulate RegIIIγ expression in IEC upon infection, confirming earlier reports that suggested an IEC-intrinsic role for MyD88 in regulating RegIIIγ expression [74, 75]. Interestingly, we did not observe significant changes in the composition of the microbiota after epithelial specific expression of MyD88 in IEC-MyD^ON^ mice. This finding is in line with our results showing that under steady-state conditions, RegIIIγ expression and epithelial permeability levels are comparable among all genotypes. Together, this suggests that under the conditions of our animal facility, the enhanced resistance of the IEC-MyD^ON^ mice is a direct consequence of functional MyD88 signaling in IEC upon infection, and not due to prior adaptations during the steady state.

Our experiments demonstrated that IEC-specific MyD88 reconstitution did not affect IL-22 production from ILC3 upon infection, indicating that the enhanced expression of RegIIIγ is not exclusively dependent on ILC3 activation. It is nevertheless possible that the basal levels of IL-22 produced by ILC3 in infected IEC-MyD^ON^ mice are still necessary to license MyD88-sufficient IEC for the induction of RegIIIγ expression. Likewise, SAA1 expression in IEC is regulated by MyD88 in an IEC-intrinsic manner during infection with *C*. *rodentium*. SAA1 has direct bactericidal effects on Gram- bacteria such as *E*. *coli* [76] and therefore may directly contribute to the host response against *C*. *rodentium*. Moreover, it has been demonstrated that attachment of segmented filamentous bacteria to epithelial cells triggers the expression of SAA1 in epithelial cells, leading to the activation of intestinal Th17 cells [48, 77]. We have observed here that reactivation of MyD88 in IEC also led to a slight but significant increase in the frequencies of Th17 cells. Whether this increase in Th17 cells results from enhanced SAA1-production in infected IEC-MyD^ON^ mice remains unclear. Nevertheless, the increase in IL-17 production may directly contribute to the enhanced barrier integrity that we observed in our system [14, 15]. Together, our data suggests that IEC-intrinsic MyD88 triggering can contribute significantly to infection resistance by enforcing the epithelial barrier function, whereas DC-specific MyD88 signaling is critical and sufficient to induce both ILC3 and Th17 cell responses upon infection with *C*. *rodentium*. It is thus likely that signals induced by MyD88 triggering in IEC and DC cooperate to induce the full host response during intestinal infections.

## Materials and methods

### Animal models

MyD^OFF^ mice [31] were crossed to CD11c-Cre [32], LysM-Cre [33], Villin-Cre [47], or Rorc-Cre [43] mice to generate cell type-specific MyD^ON^ mice. All mice were kept on C57BL/6 background and bred and maintained under specific pathogen-free conditions in our animal facilities (TWINCORE, Hannover, Germany; or Helmholtz Center for Infection Research Braunschweig, Germany). For all experiments, 8- to 14-week old mice with gender matched littermates were used.

### Ethics statement

All animal experiments were performed in compliance with the German animal protection law, Tierschutzgesetz (TierSchG, BGBl. I S. 1206, 1313, 2006/05/18). All mice were housed and handled according to good animal practice as defined by FELASA (Federation of European Laboratory Animal Science Associations) and the national animal welfare body GV-SOLAS (Gesellschaft fur Versuchstierkunde/Society for Laboratory Animal Science). All animal experiments were approved by the Lower Saxony Committee on the Ethics of Animal Experiments as well as the responsible state office (Lower Saxony State Office of Consumer Protection and Food Safety) under the permit numbers 33.9-42502-04-10/0244 and 33.9-42502-04-13/1253.

### *C*. *rodentium* infection and colony counts

*C*. *rodentium* ICC180 [78] was obtained from S. Wiles and mouse infections were performed as described previously [16]. In brief, 2 x 10^9^ bacteria were applied in 100 μl PBS intragastrically by gavage. For bacterial burden in colonic feces, stools from infected mice were collected into a pre-weighed Luria Bertani medium containing tube. Feces were then weighed, homogenized, and titrated. Series of fecal dilution were added on MacConkey Agar containing kanamycin and then cultured at 37°C for 1–2 days before counting. Bacterial burden was calculated after normalization to the weight of stool. For bacterial burden in livers, organs were homogenized in 1ml LB medium. Homogenates were further titrated, plated on MacConkey Agar containing kanamycin, and incubated at 37°C for 1–2 days before counting.

### Histopathological scoring

For histological analysis, 5 μm sections of fixed and paraffin-embedded colons were stained with haematoxilin and eosin (H&E) and examined in a blinded manner. Tissue sections were assessed for epithelial hyperplasia (score based on percentage above the height of the control, 0 = no change, 1 = 1–50%, 2 = 51–99%, 3 = 100%) and mononuclear cell infiltration (0 = none, 1 = mild, 2 = moderate, 3 = severe). Maximum score was 6. Samples were imaged under a microscope (Carl Zeiss) and processed with Nuance software 2.10.0 (Carl Zeiss).

### Isolation of colonic epithelial cells and lamina propria cells

IEC and cLP cells were isolated as described previously [16]. In brief, colon was incubated with 30 mM EDTA in PBS and then washed extensively in PBS to isolate epithelial cells. Remaining tissue was cut into small pieces and digested in several rounds with 1 mg/ml Collagenase D and 0.1 mg/ml DNase (both from Roche). Cells were enriched using 40%/80% Percoll (GE Healthcare) gradient and subsequently used for *in vitro* restimulation or flow cytometry analysis.

### Flow cytometry

Monoclonal antibodies specific to the following mouse antigens and labeled with the indicated fluorescent markers were used: CD3e-FITC and CD3e-APC (145-2C11), CD3e-APC-eFluor780 (17A2), CD4-eFluor450 (RM4-5), CD11b-APC, CD11b-APC-eFluor780, CD11b-eFluor450 (M1/70), CD11c-Alexa488, CD11c-APC-eFluor780 and CD11c-eFluor660 (N418), CD19-APC (eBio1D3), CD26-PerCP-Cyanine5.5 (H194-112), CD45R/B220-Alexa647 (RA3-6B2), CD90.2-PE-Cy7 (53–2.1), F4/80-eFluor450 (BM8), Gr-1(Ly-6G)-eFluor660 (RB6-8C5), IFN-γ-FITC and IFN-γ-PE-Cy7 (XMG1.2), IL-17A-APC (eBio17B7), IL-22-PE and IL-22-PerCP-eFluor710 (1H8PWSR), MHC-II-Biotin (2G9), MHC-II-FITC (M5/114.15.2), NK1.1-APC (PK136), rat IgG1 kappa isotype control (eBRG1), RORγt-APC and RORγt-PE (B2D), TCRβ-APC (H57-597), TCRγδ-APC (eBioGL3) and Ter119-APC (TER-119) were purchased from eBioscience/Thermo Fisher Scientific. CD64-APC, CD64-PE and CD64-BV421 (X54-5/7.1), CD103-Pacific Blue (2E7), CD172a-PE-Cy7 (P84), F4/80-Alexa700 (BM8), Ly-6C-PE-Cy7 (HK1.4), Ly-6G-Biotin (1A8) and XCR1-BV421 (ZET) were purchased from Biolegend. CD45-PE/Texas-red (30-F11) was purchased from Invitrogen/Thermo Fisher Scientific and RORγt-BV421 (Q31-378) was purchased from BD Biosciences. Dead cells were excluded by LIVE/DEAD Fixable Dead Cell Stain Kit (Life Technologies/Thermo Fisher Scientific). For intracellular cytokine staining, cells were stimulated *in vitro* with 1 μg/ml ionomycin and 0.1μg/ml phorbol-12-myristate-13-acetate (both from Sigma-Aldrich) for 2 h followed by 5 μg/ml Brefeldin A (Sigma-Aldrich) for 2 h and stained using Foxp3/Transcription Factor Fixation/Permeabilization Kit (Affymetrix/eBioscience) according to the manufacturer’s instructions. For IL-22 staining, stimulation was performed in the presence of 40 ng/ml rm-IL-23 (R&D) and following 4 h incubation with Brefeldin A. Cells were acquired on a LSR II (BD) and data were analyzed with FlowJo software (Tree Star).

### Gene expression analysis

RNA from IEC was isolated using TRIzol and transcribed into cDNA using SuperScript III Reverse Transcriptase Kit (both from Thermo Fisher Scientific). Real-time PCR was performed using iQ SYBR Green Supermix (Bio-Rad) on a LightCycler 480 II (Roche). All procedures were performed according to the manufacturer’s instructions. For expression analysis of *Saa1*, RT^2^ qPCR Primer Assays (Qiagen) were used. Primers for *Actb*, *Cxcl1*, *Duox2*, *Duoxa2*, *Muc2*, *Myd88*, *Nos2* and *RegIIIγ* were obtained from Eurofins MWG Operon with following sequences: *Actb* fwd 5’-TGTTACCAACTGGGACGACA-3’, *Actb* rev 5’-GGGGTGTTGAAGGTCTCAAA-3’, *Cxcl1* fwd 5’-CCGAAGTCATAGCCACACTCAA-3’, *Cxcl1* rev 5’-GCAGTCTGTCTTCTTTCTCCGTTAC-3’, *Duox2* fwd 5’-ACCGCTGCTCATTGTTATCC-3’, *Duox2* rev 5’-AGTGCACAGCCACAATTTCG-3’, *Duoxa2* fwd 5’-TTCAGCACATCTGCAGACAC-3’ and *Duoxa2* rev 5’-AGCCTGAAGTCATGTTTGCC-3’. *Muc2* fwd 5’-ATGCCCACCTCCTCAAAGAC-3’, *Muc2* rev 5’-GTAGTTTCCGTTGGAACAGTGAA-3’, *Myd88* fwd 5’-TGGCCTTGTTAGACCGTGA-3’, *Myd88* rev 5’-AAGTATTTCTGGCAGTCCTCCTC-3’, *Nos2* fwd 5’-AGAGCCAAGCTGGAAGATACAC-3’, *Nos2* rev 5’-ATCAAGGTGGCGTCTCTCTG-3’, *Reg3g* fwd 5’-CCTTCCTCTTCCTCAGGCAAT-3_,_ *Reg3g* rev 5’-TAATTCTCTCTCCACTTCAGAAATCCT-3’, Gene expression was normalized to *Actb* and log_2_ transformed.

To determine gene expression in colonic DC/MO populations, DC/MO were sorted on a FACS ARIA Fusion (BD) directly into RLT buffer (Qiagen) and total RNA was isolated using RNeasy Micro Kit (Qiagen) according to the manufacturer’s instructions. RNA quality and quantity was assessed using Agilent 2100 Bioanalyzer (Agilent Technologies). 200–500 pg of high quality total RNA was subjected to one linear mRNA amplification cycle using the MessageBooster kit for quantitative RT-PCR (Epicentre). 50–100 ng of amplified mRNA was transcribed into cDNA using SuperScript III Reverse Transcriptase Kit (Thermo Fisher Scientific). All procedures were performed according to the manufacturer’s protocols. Real-time PCR was performed using iQ SYBR Green Supermix (Bio-Rad) with RT^2^ qPCR Primer Assays (Qiagen). Gene expression was normalized to five housekeeping genes (*Actb*, *B2m*, *Gapdh*, *Gusb*, *Hsp90ab1)* and log2 transformed and is represented as bar graphs or heat maps using Cluster 3.0 and JavaTree software.

### Intestinal permeability assay

The FITC-dextran intestinal permeability assay was adapted from the method described by Gupta et al. [79]. Infected mice were gavaged on day 8 p.i. with 100 μl of 44 mg/100 g body weight FITC- dextran (4-kDa, Sigma-Aldrich) in PBS 4 h prior to sacrifice. Blood was collected by cardiac puncture and serum was separated by centrifugation (13.000 rpm, 10 min). FITC-dextran concentration in serum was determined by spectrophotofluorometry with an excitation of 485 nm (20 nm band width) and an emission wavelength of 528 nm (20 nm band width) on an ELx800 Reader (BioTEK), and standard serially diluted FITC-dextran was used to calculate final serum concentrations. Serum from mice not administered with FITC-dextran was used to determine the background.

### Microbiota composition analysis

Fresh fecal pellets were collected and immediately stored at -20°C. DNA was extracted using a method combining bead-beating and phenol/chloroform-based purification as described previously [80]. Briefly, 200 μl of 0.1-mm diameter zirconia/silica beads, 500 μl of extraction buffer (200 mM Tris, 20mM EDTA, 200mM NaCl, pH 8.0), 200μl of 20% SDS and 500μl of phenol:chloroform:isoamyl alcohol (24:24:1) was added to frozen samples. To lyse bacterial cells, mechanical disruption was performed using a Mini-BeadBeater-96 (BioSpec) and samples were homogenized twice for 2 min. After DNA isolation, DNA was resuspended in TE buffer with 100 μg/ml RNAse. For 16S rRNA sequencing, samples were purified using spin columns (BioBasic) and normalized to 25 ng/μl. Amplification of the V4 region (F515/R806) of the 16S rRNA gene was performed using a common forward primer and a barcoded reverse primer according to previously described protocols [81]. Up to 584 samples were pooled and sequenced on an Illuminia MiSeq platform (PE250). Obtained sequences were filtered for low quality reads and binned based on sample-specific barcodes using QIIME v1.8.0 [82]. Reads were clustered into 97% ID OTUs using UCLUST, followed by taxonomic classification using the RDP Classifier executed at 80% bootstrap confidence cut off [83, 84]. Sequences without matching reference dataset, were grouped as *de novo* using UCLUST. Phylogenetic relationships between OTUs are determined using FASTTREE to the PyNAST alignment [85]. The OTU absolute abundance table and mapping file are used for statistical analyses and data visualization in the R statistical programming environment package PHYLOSEQ [86].

### Statistical analysis

Data analysis was performed using GraphPad Prism Software 5.0. Statistics were calculated using One-Way ANOVA with Bonferroni’s Multiple Comparison test (*p<0.05, **p<0.01 and ***p<0.001), unless otherwise stated in the Figure legends.

## Supporting information

S1 Fig Cell type-specific targeting in CD11c-Cre and LysM-Cre driven genetic approaches. (A) Crossing procedure to generate RFP^ON^ mice. ROSA26-tdRFP mice were described previously [1] and crossed to CD11c-Cre and LysM-Cre mice, respectively. RFP^ON^ mice were analyzed for the targeting efficiency of intestinal MNP by the Cre-driven approaches. (B) Representative flow cytometry data illustrating RFP expression (red histograms) in colonic MO (gated on single lin^−^MHC-II^+^CD64^+^F4/80^+^ cells) and DC (gated on single lin^−^MHC-II^+^CD64^−^CD26^+^CD11c^+^) amongst live cLP cells. Grey histograms represents signal in RFP-WT littermate. Bar graphs show targeting efficiency of colonic MO and conventional XCR1^+^ cDC1 and CD172a^+^ cDC2 in CD11c-RFP^ON^ and LysM-RFP^ON^ mice. Data were pooled from two independent experiments with n = 4 mice. Error bar represents +SEM.
(TIF)

S2 Fig Gating strategy for the identification of colonic ILC3. Representative flow cytometry plots illustrating the gating strategy for ILC3 in cells isolated from the cLP. ILC3 were gated as single, live lin^−^CD90^+^RORγt^+^ cells. As lineage marker, antibodies against TCRβ, TCRγδ, CD19, Gr-1, Ter119, NK1.1, CD11c and CD11b were included.
(TIF)

S3 Fig Restoring MyD88 signaling in CD11c^+^ cells increases the frequencies of IL-17 -producing ILC3 in the colon of *C. rodentium* infected mice. Leukocytes were isolated from the cLP of mice before (control) and on day 4 p.i. (infected) with *C*. *rodentium* and analyzed by flow cytometry. Representative flow cytometry plots showing the frequency of IL-17^+^ cells within live ILC3. Data were pooled from 3 independent experiments n = 2–5 mice per group. One-Way ANOVA with Bonferroni’s Multiple Comparison test, *p<0.05, **p<0.01, ns–not significant.
(TIF)

S4 Fig Colons of WT, MyD^OFF^, CD11c-MyD^ON^ and LysM-MyD^ON^ mice show a normal, healthy appearance during steady-state conditions. Representative H&E staining of colon sections from WT, MyD^OFF^, CD11c-MyD^ON^ and LysM-MyD^ON^ mice before infection with *C*. *rodentium*. Scale bar represents 100 μm.
(TIF)

S5 Fig Frequencies and total numbers of neutrophils, MO and DC in the colon of mice infected with *C. rodentium*. Leukocytes were isolated from the cLP of mice on day 8 p.i. and analyzed by flow cytometry. Representative flow cytometry plots showing the frequencies of neutrophils (live single CD64^−^Ly6G^+^Ly6C^int^), MO (live single CD11b^+^CD64^+^) and DC (live single CD64^−^CD11c^+^MHC-II^+^). Graphs represent frequency (%) and total number (#) of neutrophils, MO and DC (lower panel). Data shown for one experiment out of two with n = 3–6 mice per group. Error bar represents +SEM. One-Way ANOVA with Bonferroni’s Multiple Comparison test; *p<0.05, **p<0.01, ***p<0.001.
(TIF)

S6 Fig Restoring MyD88 signaling in CD11c^+^ cells increases the total number of cytokine-producing T cells in the colon of *C. rodentium* infected mice. Leukocytes were isolated from the cLP of mice before (control) or on day 8 p.i. (infected) with *C*. *rodentium* and the T cell response was analyzed by flow cytometry. Graphs represent total number (#) of IL-17A^+^, IFN-γ^+^ and IL-22^+^ cells amongst live CD3^+^CD4^+^ T cells. Data were pooled from 2 independent experiments with n = 3–5 mice per group. Error bar represents +SEM. One-Way ANOVA with Bonferroni’s Multiple Comparison test; *p<0.05, **p<0.01.
(TIF)

S7 Fig Gating strategy for the isolation of colonic DC and MO by FACS. Representative flow cytometry plots illustrating the gating strategy for sorting of DC and MO from the cLP of WT, MyD^OFF^, CD11c-MyD^ON^ and LysM-MyD^ON^ mice on day 4 p.i. with *C*. *rodentium*. DC were sorted as live single CD64^−^F4/80^−^lin^−^MHC-II^+^CD26^+^CD11c^+^ and MO as live single CD64^+^F4/80^+^lin^−^MHC-II^+^ cells. As lineage marker, antibodies against CD3, CD19, B220 and NK1.1 were included. Post-sort analysis confirmed a purity of >98%.
(TIF)

S8 Fig Targeting of colonic T cells and ILC3 in CD11c-RFP^ON^ mice. Representative histogram showing RFP expression amongst colonic live CD3^+^CD4^+^ T cells (A) and ILC3 (B) in infected CD11c-RFP^ON^ mice on day 8 (A) and day 4 (B) p.i. (black histogram). Tinted grey histogram represents signal in RFP-WT littermate.
(TIF)

S9 Fig Reactivation of *Myd88* expression in IEC from IEC-MyD^ON^ mice. *Myd88* gene expression in IEC isolated on day 4 p.i. with *C*. *rodentium* from the colon of WT, MyD^OFF^ and IEC-MyD^ON^ mice. Data shown as mean relative expression to *Actb*+SEM. Data were pooled from three individual experiments with n = 3 mice per group.
(TIF)

S10 Fig Composition of the colonic microbiota before (control) and 4 days p.i. (infection) with *C. rodentium*. Relative abundances of bacterial families are shown and grouped according to their phylum. Bars represent mean of all mice within the group with n = 5–11 mice per group.
(TIF)

S1 References (DOCX)
