# Supplementary figures and images for: MyD88 signaling in dendritic cells and the intestinal epithelium controls immunity against intestinal infection with C. rodentium
Source: PLoS Pathog. 2017 May 16;13(5):e1006357. doi: 10.1371/journal.ppat.1006357 (PMC5433783; doi:10.1371/journal.ppat.1006357)

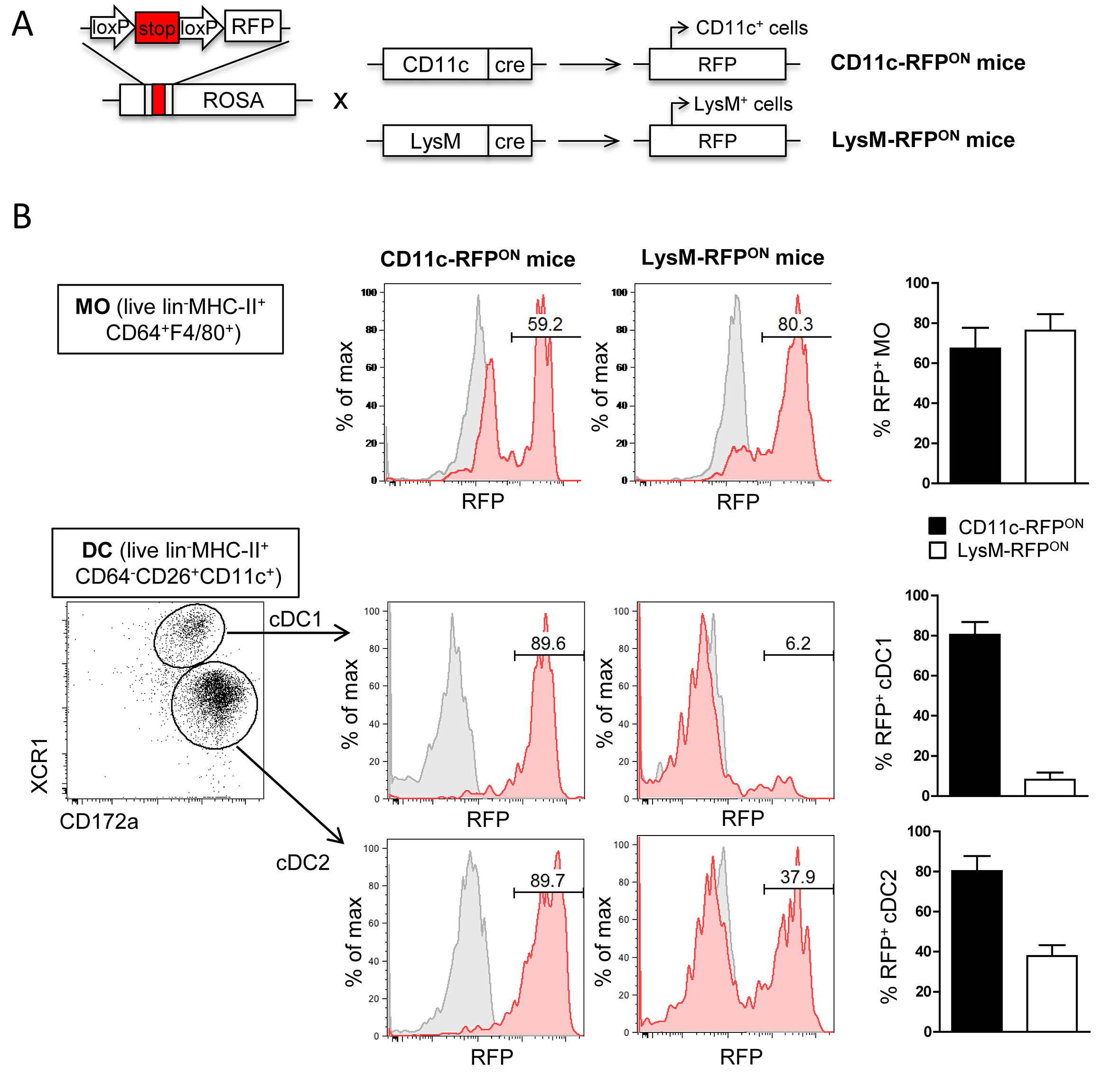

Supplement: S1 Fig — (A) Crossing procedure to generate RFPON mice. ROSA26-tdRFP mice were described previously [1] and crossed to CD11c-Cre and LysM-Cre mice, respectively. RFPON mice were analyzed for the targeting efficiency of intestinal MNP by the Cre-driven approaches. (B) Representative flow cytometry data illustrating RFP expression (red histograms) in colonic MO (gated on single lin−MHC-II+CD64+F4/80+ cells) and DC (gated on single lin−MHC-II+CD64−CD26+CD11c+) amongst live cLP cells. Grey histograms represents signal in RFP-WT littermate. Bar graphs show targeting efficiency of colonic MO and conventional XCR1+ cDC1 and CD172a+ cDC2 in CD11c-RFPON and LysM-RFPON mice. Data were pooled from two independent experiments with n = 4 mice. Error bar represents +SEM. (TIF) [file ppat.1006357.s001.tif]

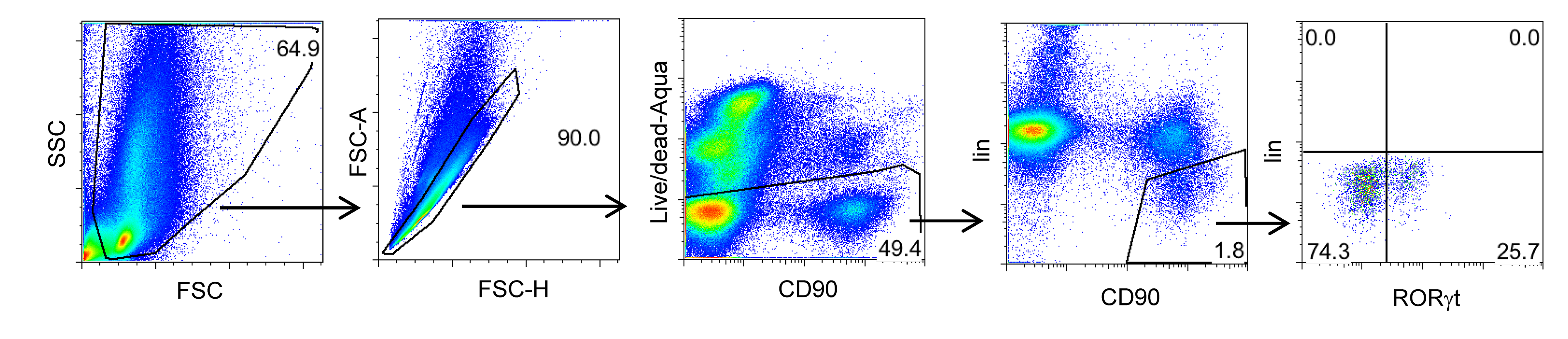

Supplement: S2 Fig — Representative flow cytometry plots illustrating the gating strategy for ILC3 in cells isolated from the cLP. ILC3 were gated as single, live lin−CD90+RORγt+ cells. As lineage marker, antibodies against TCRβ, TCRγδ, CD19, Gr-1, Ter119, NK1.1, CD11c and CD11b were included. (TIF) [file ppat.1006357.s002.tif]

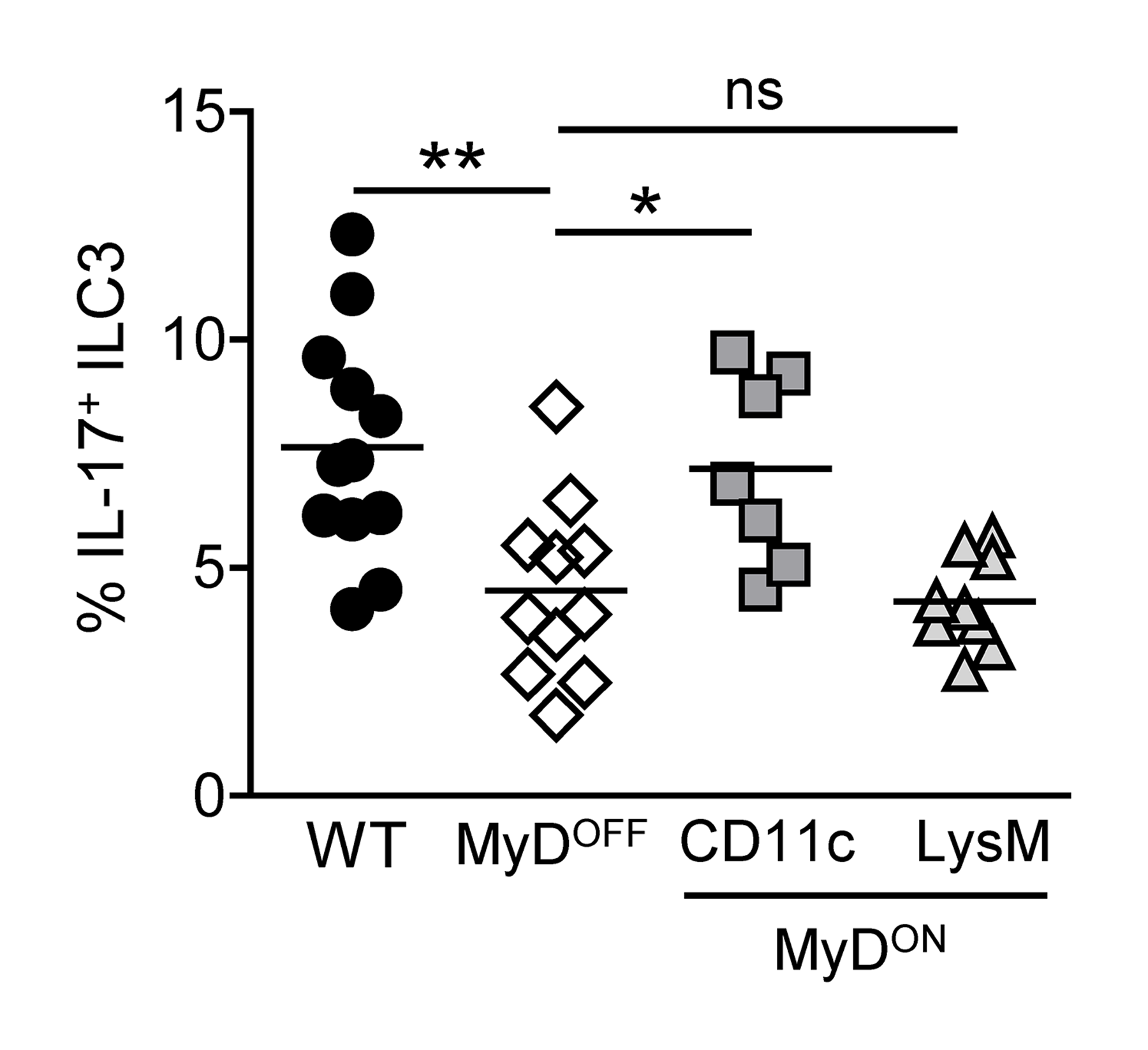

Supplement: S3 Fig — Leukocytes were isolated from the cLP of mice before (control) and on day 4 p.i. (infected) with C. rodentium and analyzed by flow cytometry. Representative flow cytometry plots showing the frequency of IL-17+ cells within live ILC3. Data were pooled from 3 independent experiments n = 2–5 mice per group. One-Way ANOVA with Bonferroni’s Multiple Comparison test, *p<0.05, **p<0.01, ns–not significant. (TIF) [file ppat.1006357.s003.tif]

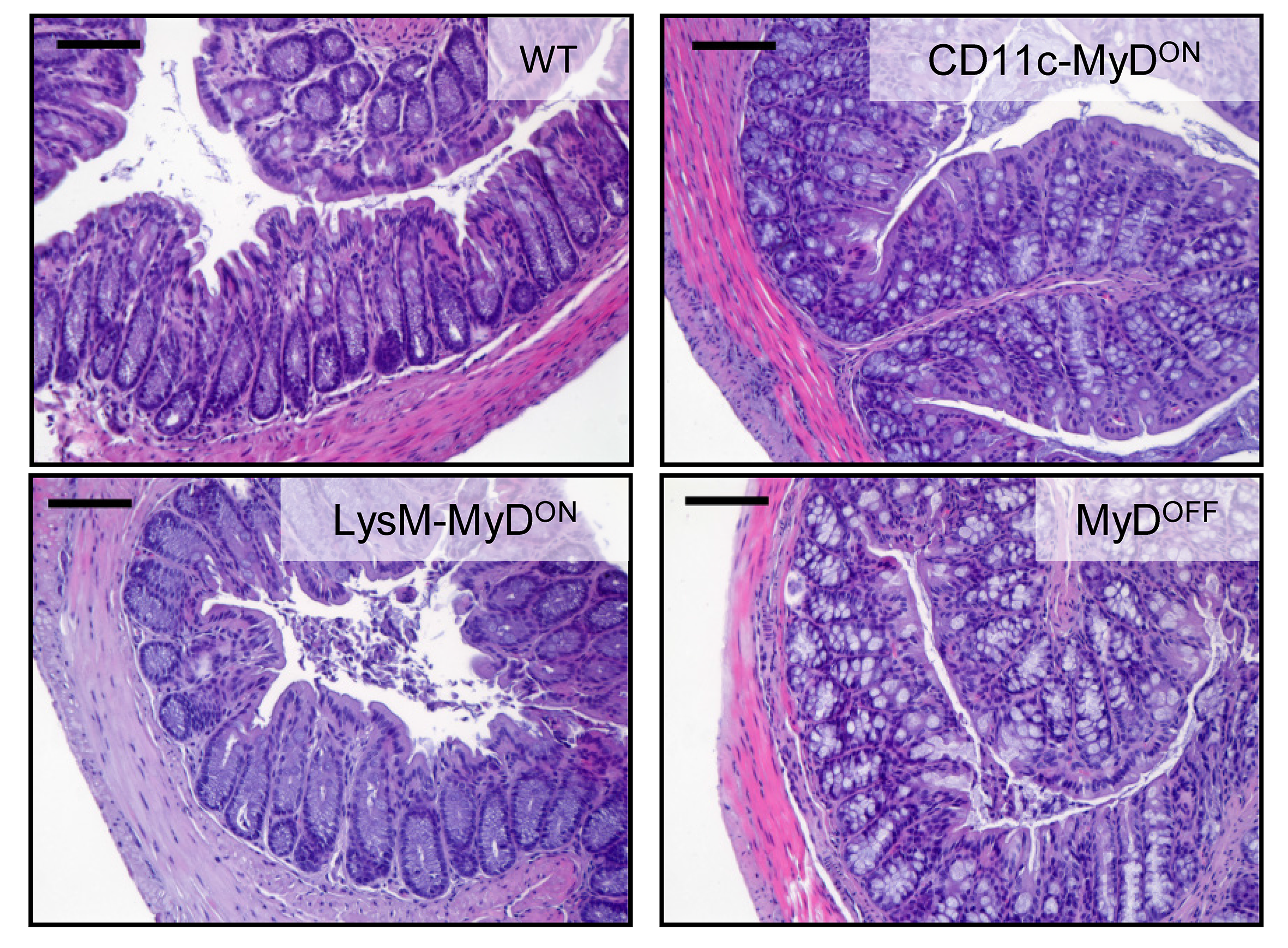

Supplement: S4 Fig — Representative H&E staining of colon sections from WT, MyDOFF, CD11c-MyDON and LysM-MyDON mice before infection with C. rodentium. Scale bar represents 100 μm. (TIF) [file ppat.1006357.s004.tif]

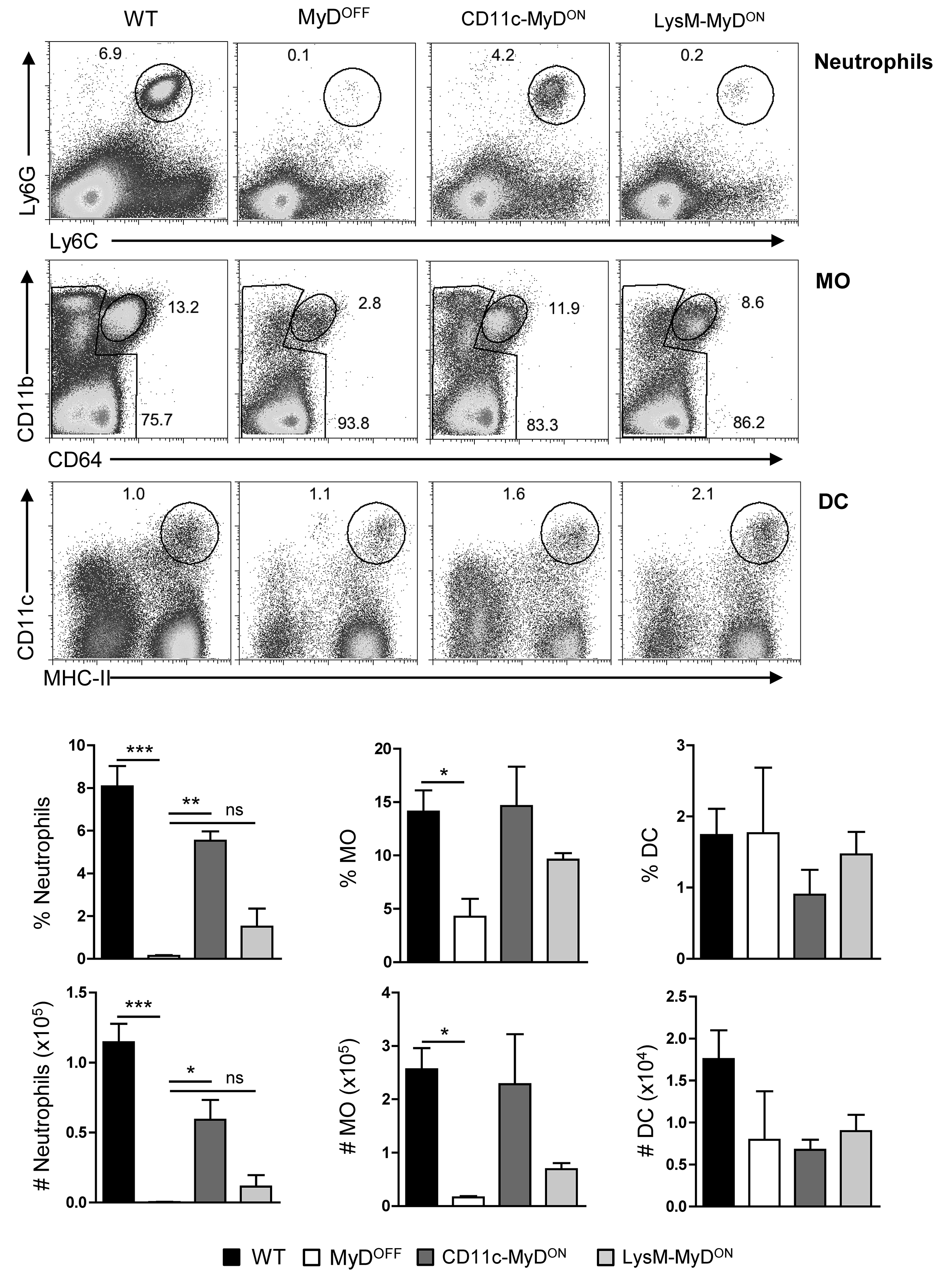

Supplement: S5 Fig — Leukocytes were isolated from the cLP of mice on day 8 p.i. and analyzed by flow cytometry. Representative flow cytometry plots showing the frequencies of neutrophils (live single CD64−Ly6G+Ly6Cint), MO (live single CD11b+CD64+) and DC (live single CD64−CD11c+MHC-II+). Graphs represent frequency (%) and total number (#) of neutrophils, MO and DC (lower panel). Data shown for one experiment out of two with n = 3–6 mice per group. Error bar represents +SEM. One-Way ANOVA with Bonferroni’s Multiple Comparison test; *p<0.05, **p<0.01, ***p<0.001. (TIF) [file ppat.1006357.s005.tif]

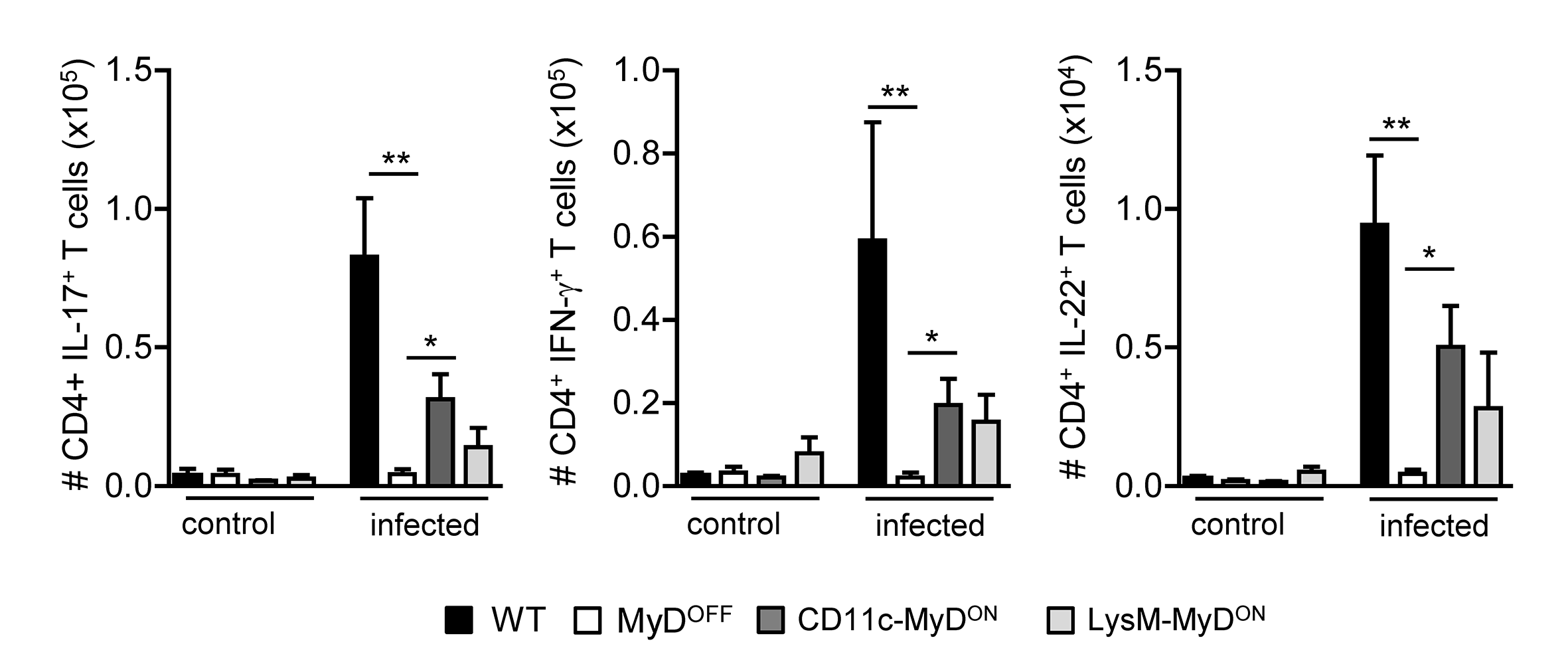

Supplement: S6 Fig — Leukocytes were isolated from the cLP of mice before (control) or on day 8 p.i. (infected) with C. rodentium and the T cell response was analyzed by flow cytometry. Graphs represent total number (#) of IL-17A+, IFN-γ+ and IL-22+ cells amongst live CD3+CD4+ T cells. Data were pooled from 2 independent experiments with n = 3–5 mice per group. Error bar represents +SEM. One-Way ANOVA with Bonferroni’s Multiple Comparison test; *p<0.05, **p<0.01. (TIF) [file ppat.1006357.s006.tif]

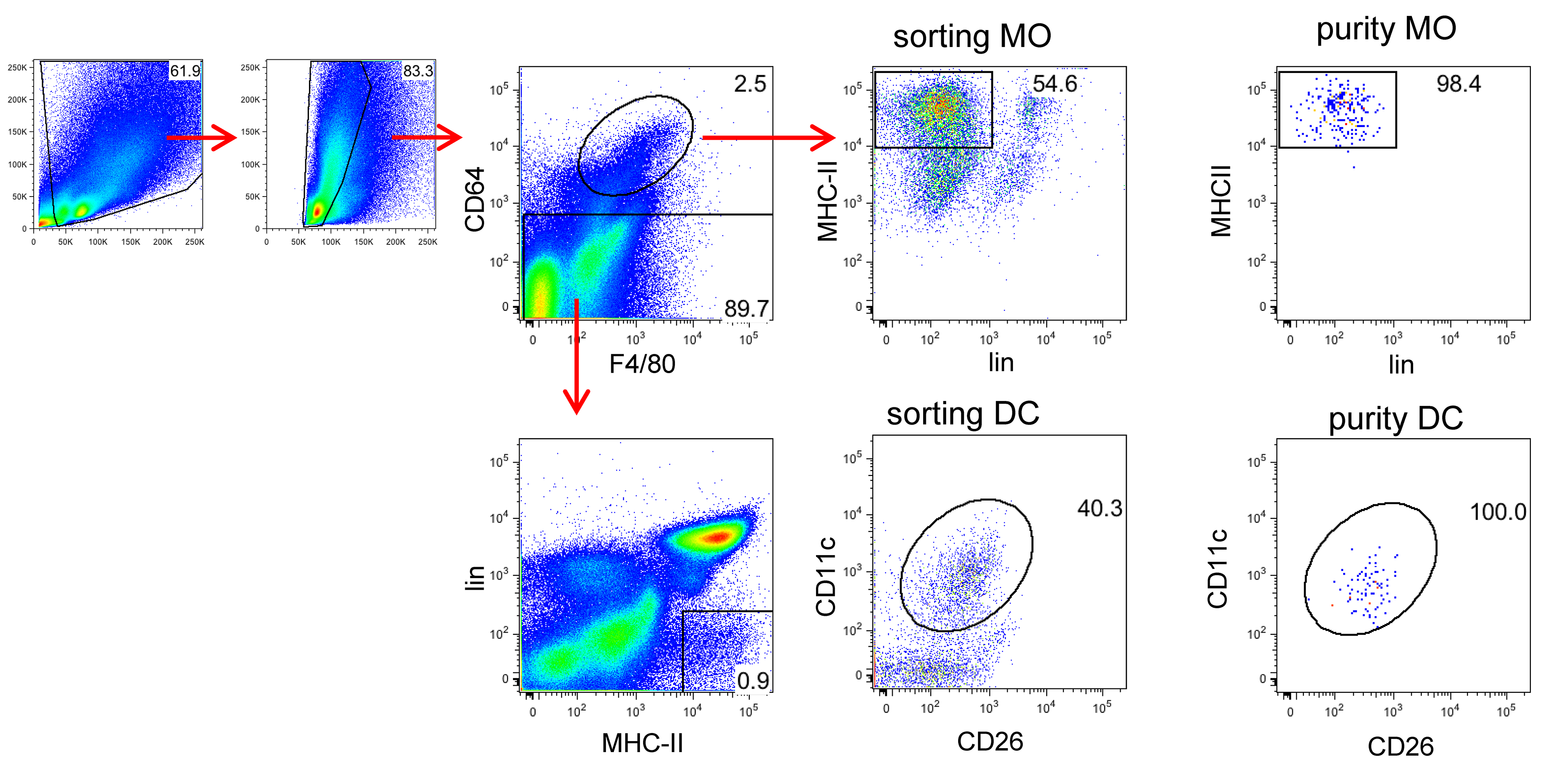

Supplement: S7 Fig — Representative flow cytometry plots illustrating the gating strategy for sorting of DC and MO from the cLP of WT, MyDOFF, CD11c-MyDON and LysM-MyDON mice on day 4 p.i. with C. rodentium. DC were sorted as live single CD64−F4/80−lin−MHC-II+CD26+CD11c+ and MO as live single CD64+F4/80+lin−MHC-II+ cells. As lineage marker, antibodies against CD3, CD19, B220 and NK1.1 were included. Post-sort analysis confirmed a purity of >98%. (TIF) [file ppat.1006357.s007.tif]

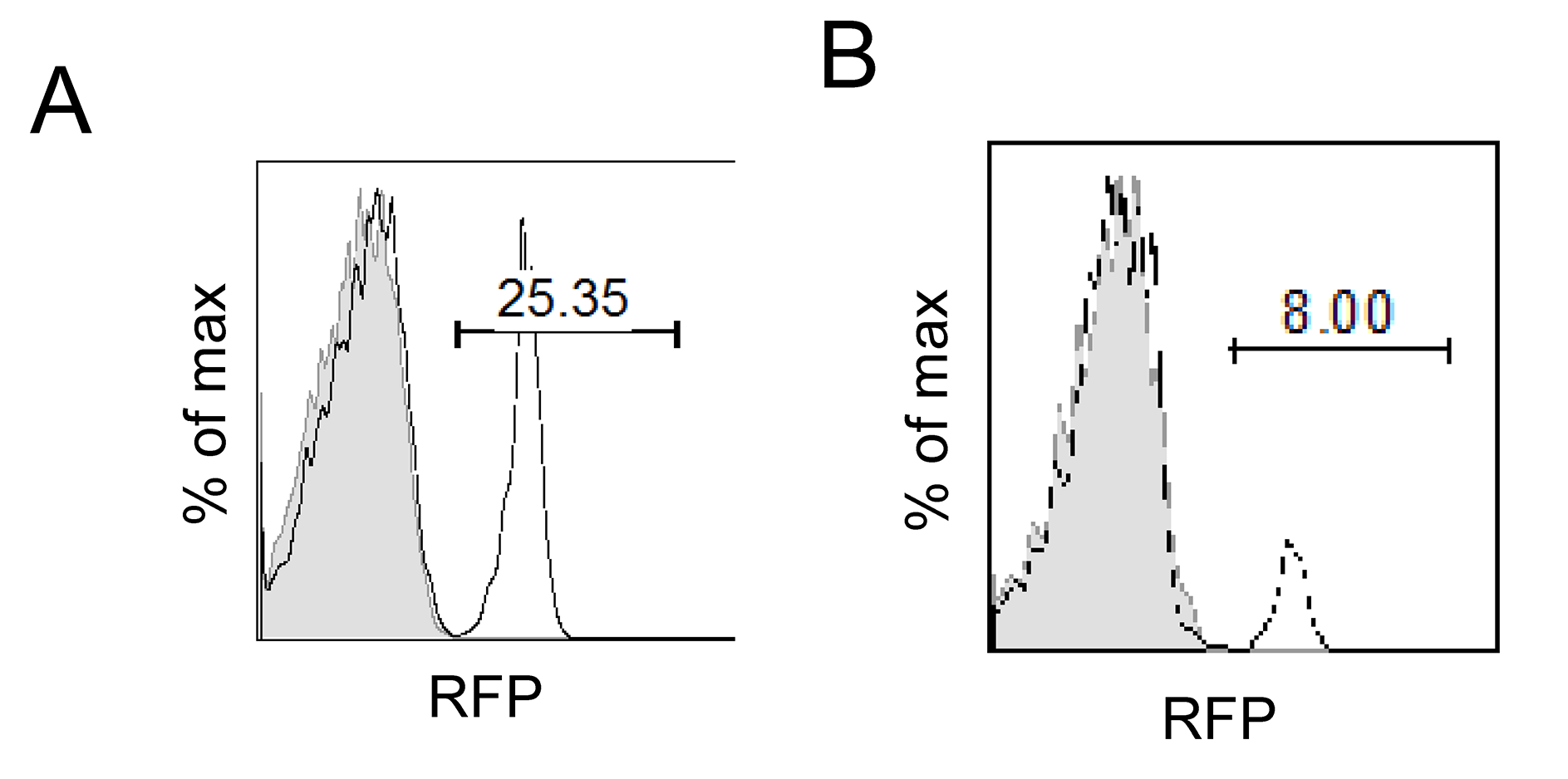

Supplement: S8 Fig — Representative histogram showing RFP expression amongst colonic live CD3+CD4+ T cells (A) and ILC3 (B) in infected CD11c-RFPON mice on day 8 (A) and day 4 (B) p.i. (black histogram). Tinted grey histogram represents signal in RFP-WT littermate. (TIF) [file ppat.1006357.s008.tif]

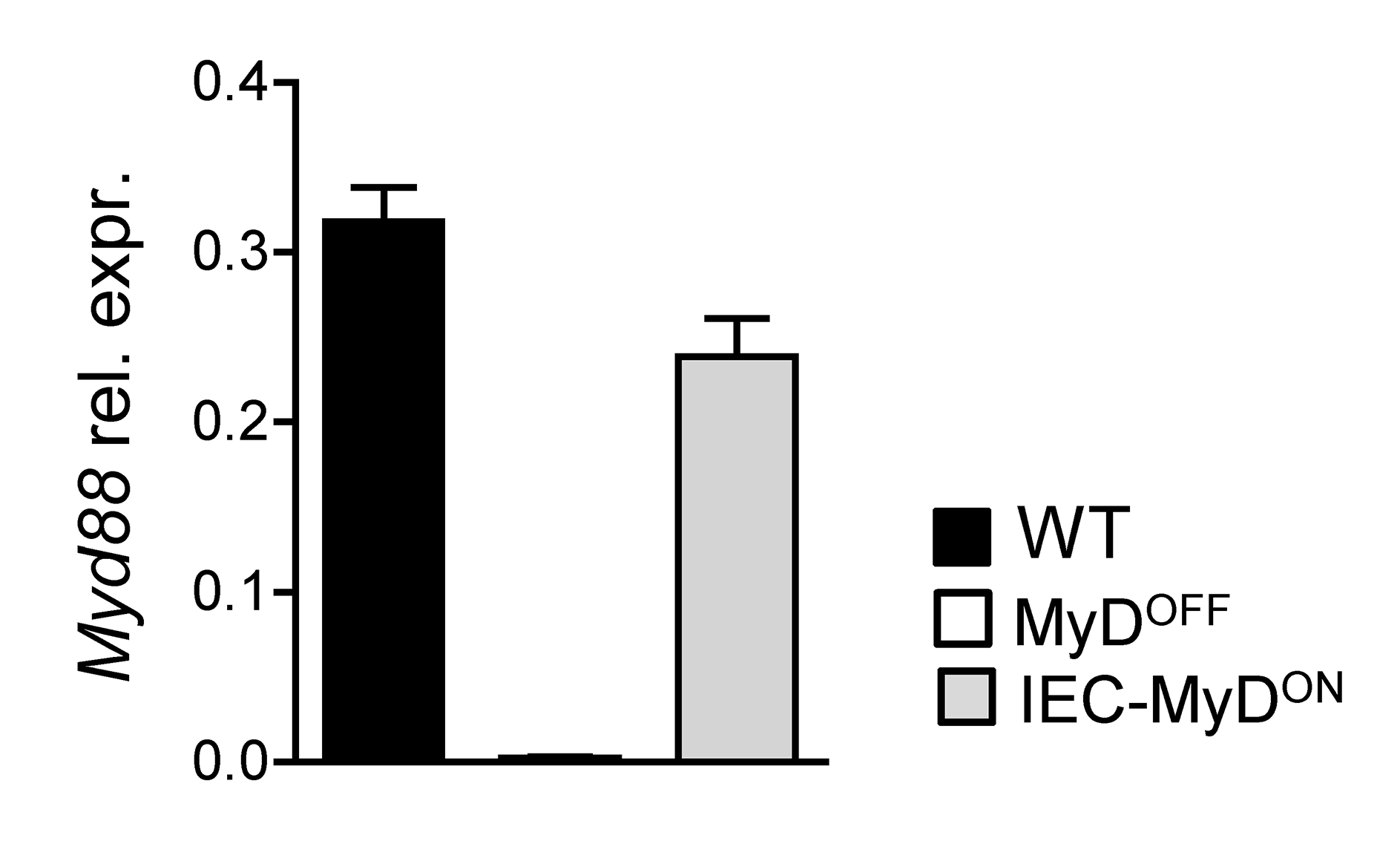

Supplement: S9 Fig — Myd88 gene expression in IEC isolated on day 4 p.i. with C. rodentium from the colon of WT, MyDOFF and IEC-MyDON mice. Data shown as mean relative expression to Actb+SEM. Data were pooled from three individual experiments with n = 3 mice per group. (TIF) [file ppat.1006357.s009.tif]

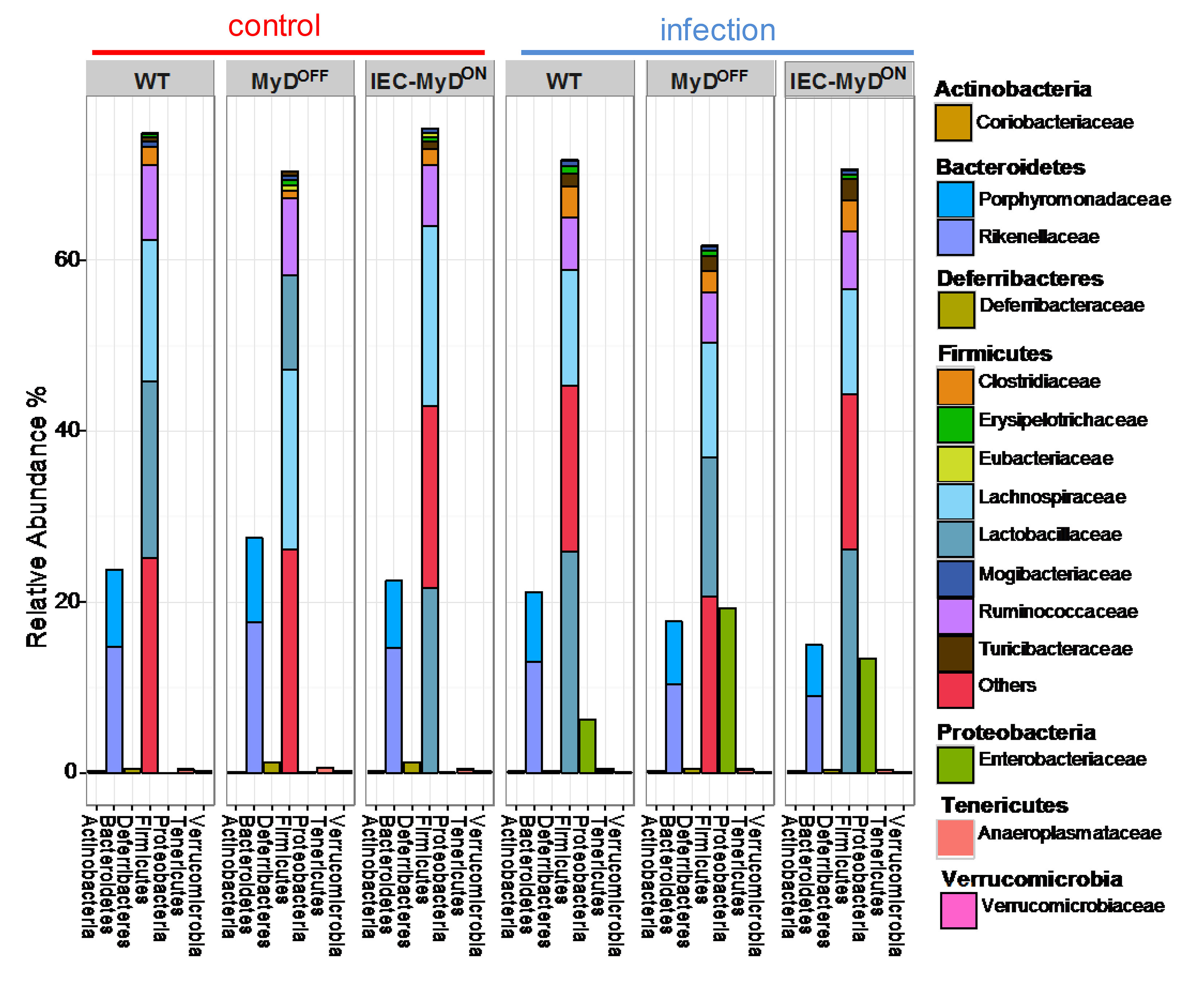

Supplement: S10 Fig — Relative abundances of bacterial families are shown and grouped according to their phylum. Bars represent mean of all mice within the group with n = 5–11 mice per group. (TIF) [file ppat.1006357.s010.tif]
