# Supplementary material for: MyD88 signaling in dendritic cells and the intestinal epithelium controls immunity against intestinal infection with C. rodentium
Source: PLoS Pathog. 2017 May 16;13(5):e1006357. doi: 10.1371/journal.ppat.1006357 (PMC5433783; doi:10.1371/journal.ppat.1006357)
Supplement: S1 References — (DOCX) [file ppat.1006357.s011.docx]

**S1 References**

1. Luche H, Weber O, Nageswara Rao T, Blum C, Fehling HJ. Faithful activation of an extra-bright red fluorescent protein in "knock-in" Cre-reporter mice ideally suited for lineage tracing studies. Eur J Immunol. 2007;37(1):43-53.
